# Unveiling the multifunctional use of ochre in the Middle Stone Age: Specialized ochre retouchers from Blombos Cave

**DOI:** 10.1126/sciadv.ads2797

**Published:** 2025-06-27

**Authors:** Elizabeth C. Velliky, Francesco d’Errico, Karen L. van Niekerk, Christopher S. Henshilwood

**Affiliations:** ^1^ Centre for Early Sapiens Behaviour (SapienCE), Department of Archaeology, History, Cultural Studies and Religion, University of Bergen, Bergen, Norway.; ^2^ Université de Bordeaux, PACEA UMR 5199, CNRS, Pessac, France.; ^3^ Archaeology Divion, School of Geography, Archaeology and Environmental Studies, University of the Witwatersrand, Johannesburg, South Africa.; ^4^ Evolutionary Studies Institute, University of the Witwatersrand, Johannesburg, South Africa.

## Abstract

Ochre, an iron-rich pigment, is widely associated with symbolic communication, but its functional applications in the Middle Stone Age (MSA) remain poorly understood. Experimental and ethnographic evidence suggests ochre being useful for hide tanning, hafting adhesives, and skin protection, although direct archeological evidence is scarce. We address this gap by presenting ochre tools from Blombos Cave, South Africa, found in Still Bay to pre–Still Bay layers dated 90 to 70,000 years ago. Seven ochre pieces were deliberately modified into lithic retouchers, showing clear use-wear patterns and evidence of intentional shaping. Targeted experiments confirm that some were used for pressure flaking and were rejuvenated to maintain function. These findings provide direct evidence of ochre being used to retouch lithic artifacts during the MSA, highlighting its role in technological systems of this period. The results emphasize the multifunctionality of ochre and suggest that such curated tools may have held personal, cultural, or technological significance within early modern human communities.

## Teaser

Seven ochre artifacts from Blombos Cave show evidence of lithic retouching and pressure flaking, revealing specialized MSA tools.

## INTRODUCTION

The collection and use of iron-rich earth materials colloquially referred to by archeologists as “ochre” emerged in Africa during the Early Stone Age (*1*, *2*) and subsequently fluoresced throughout the African Middle Stone Age (MSA), spanning from roughly 300 to 30 thousand years (ka) before present (BP) (*3*, *4*). Increasing evidence shows that the recognition and subsequent use of earth pigments is not restricted to *Homo sapiens*, with several examples offering compelling evidence from Lower, Middle, and late Middle Paleolithic Eurasian sites (*5*–*8*). However, few contexts outside Africa match the intensity, variety, and abundance of ochre materials found at African MSA sites. Thus, the collection and use of ochre and earth pigments likely played a pivotal role during the early emergence and spread of distinctly modern human behaviors, colloquially referred to as behavioral modernity, including defining traits such as symbolic mediation, complex syntactical language, advanced cognition, and various cultural expressions (*9*, *10*). Although it is generally accepted that the presence of ochre and pigments at prehistoric archeological sites indicates their role as a tool for cultural expression, unexpectedly, little is understood about the intricacies of these expressions and how they materialize archeologically.

The practices in which mineral pigments may have been involved are generally labeled by archeologists as “symbolic” and “functional,” where symbolic use refers to the ways in which ochre was used to convey meaning and to participate in rituals and cultural tradition. This includes using ochre in bodily adornment, whether directly or indirectly through personal ornaments, as a pigment or paint mixture for creating rock art or using it in burials or other ceremonial contexts. Functional use refers to the practical applications of ochre, including its use as a hide-tanning agent, hafting mastic, sunscreen, and insect repellent. Although the possible range of applications of this material is vast, in most cases, the specific practices are difficult to document through archeology alone. Many functional applications are only suggested by experimental research (*11*–*14*) or ethnographic comparison. Specific symbolic practices are speculative and are often based on site-specific examples and from which it is difficult to draw ethnographic comparisons, given the dynamic and ephemeral nature of symbolic practices. Furthermore, while these categories provide a useful framework for situating the use of ochre in archeological contexts, they do not capture the full complexity of its role in MSA societies. Practices involving ochre materials likely had multiple meanings and functions and may have varied greatly, even within contemporaneous contexts (*15*, *16*). In southern Africa, the discussion of ochre and its role in human cultural evolution is continuously evolving, with more varied and complex evidence emerging from different areas, contexts, and time periods (*1*, *17*, *18*).

This paper aims to present and document findings on an artifact type not previously identified from Blombos Cave (BBC) in South Africa. Here, we identified seven artifacts made from ochre and bearing marks, possibly indicating their use in knapping activities and as pressure flakers during a qualitative assessment of the ochre assemblage. Targeted experiments confirmed that some were used as soft hammers for knapping and others were used for pressure flaking.

As ochre and earth pigments are known to have played an important role in early human cultural expression, exploring their function in tool production adds a previously unidentified dimension to our understanding of MSA ochre-related behaviors. Here, we provide evidence for ochre tool use and, through which, establish this practice as a lithic retouching technique in the pre–Still Bay layers. The presence of tools used for pressure flaking indicates a possible precursor to pressure flaking found during the Still Bay period, showcasing technological innovation and cultural continuity of this practice over time at the site. We interpret the pieces as being intentionally crafted for a specific function—lithic retouching, thus indicating their use as “specialized tools.” However, they also likely held a symbolic dimension (*19*). We define specialized tool as one conceived and shaped to fulfill a specific function, in contrast to more versatile tools that can serve multiple purposes. For instance, an unmodified flake can be considered a nonspecialized tool, as it can be used for cutting, scraping, or other tasks without prior modification. In contrast, Still Bay points, while often interpreted as spear tips, could also have been used as knives, making them more functionally flexible. In the case of the ochre retouchers we present here, their modification and use-wear patterns suggest a primary function in lithic retouching, distinguishing them as specialized tools. Last, we use the retouchers to explore the implications for technology and social dynamics within MSA communities, contributing to the discourse on behavioral modernity and cultural evolution in southern Africa.

### Archeological background

BBC is located on the Southern Cape coast ~300 km east of Cape Town, South Africa (fig. S1) and has been pivotal in documenting MSA cultural innovations related to the early onset of complex behaviors in the MSA. The site boasts well-stratified MSA depots that have been dated to ca. 100 to 72 ka BP, which are capped by a sterile aeolian sand dune layer, followed by Later Stone Age depots (fig. S2) (*20*). The MSA sequence at BBC is separated into four phases: M1, M2 upper, M2 lower, and M3. The M1 and M2 upper phases, dated to ca. 77 to 73 ka BP (*21*), are the two phases associated with the Still Bay technocomplex and boast bifacial foliate lithic points, manufactured bone tools and engraved bone artifacts, *Nassarius kraussianus* shell beads (some of which bear ochre residues) (*22*), modified ochre pieces including complex geometric engraved designs (*23*), and a silcrete flake with similarly designed crosshatched drawn ochre lines (*24*). The preceding M2 lower phase (ca. 85 to 82 ka BP) has so far yielded no characteristic Still Bay type artifacts, while the M3 phase (ca. 101 to 94 ka BP) is rich in both modified and nonmodified ochre artifacts (including some crosshatched designs) (*25*) and an ochre “toolkit,” which shows evidence for containing an ochre pigment compound in an abalone shell from about 100 ka BP (*20*).

The complex and innovative lithic technology used during the pre–Still Bay and Still Bay periods at BBC is extensively documented and analyzed (*26*, *27*). This includes the heat treatment of silcrete (*27*) and the unique shaping of foliate bifacial points (*26*), which are characteristic features of the Still Bay technocomplex. Evidence of these activities has been found at other Still Bay and pre–Still Bay sites across South Africa, including Pinnacle Point (*28*), Sibudu (*29*, *30*), Umhlatuzana (*31*), and Hollow Rock Shelter (*32*). However, there is currently limited evidence for pressure flaking during the Still Bay. The hypothesis that the Still Bay silcrete bifacial points found at BBC were produced in the final phase of their shaping by pressure flaking has been proposed on the basis of the morphology of flake scars on the archeological pieces and the experimental reproduction of the flake scars with bone pressure flakers (*27*). However, the tools that would have been used in this delicate task were not recovered from BBC, but it has been proposed that bone retouchers were used as pressure flakers from other sites (*33*). At Umhlatuzana (*31*), pressure flaking is inferred from the morphology of microflakes and flake scars. Bone tools interpreted as pressure flakers have been identified at Sibudu in late Howieson Poort and early post–Howieson Poort layers, dated at around 61 ka (*34*). The hypothesis that ochre nodules were opportunistically used as abraders and soft hammers by the knappers of Still Bay bifacial points has been proposed at Sibudu by relying on the nature, appearance, and distribution of ochre residues present on the platforms of a few shaping flakes found at the site (*29*). The actual ochre nodules that may have left the residues on the flakes, however, were not found. Currently, only BBC (*26*), Umhlatuzana (*31*), and Sibudu (*34*) provide definitive documentation for pressure flaking.

BBC is particularly rich in ochre materials and has offered a unique glimpse into the complexity and variety of ochre- and pigment-related practices during a pivotal time in human cultural evolution. It contains more than 8000 documented ochre artifacts from the 1998/1999 excavation seasons (*35*), and there is an ongoing qualitative analysis of the ochre from subsequent excavation seasons. During this recent assessment of the ochre assemblage from BBC, several unique pieces were found. Upon identifying impact scars and striations similar to those observed on bone pressure flakers from Sibudu (*31*), we determined that the first identified piece could represent a previously undocumented ochre-related practice. This discovery led to the identification of several additional pieces. To further investigate their function, we designed an experimental protocol to reproduce the observed use-wear traces and assess whether experimental results matched the archeological evidence. Here, we present ochre pieces that were intentionally shaped and used as lithic retouchers, including for pressure flaking, from the pre–Still Bay to Still Bay layers at BBC.

## RESULTS

### The ochre retouchers

**Fig. 1.**
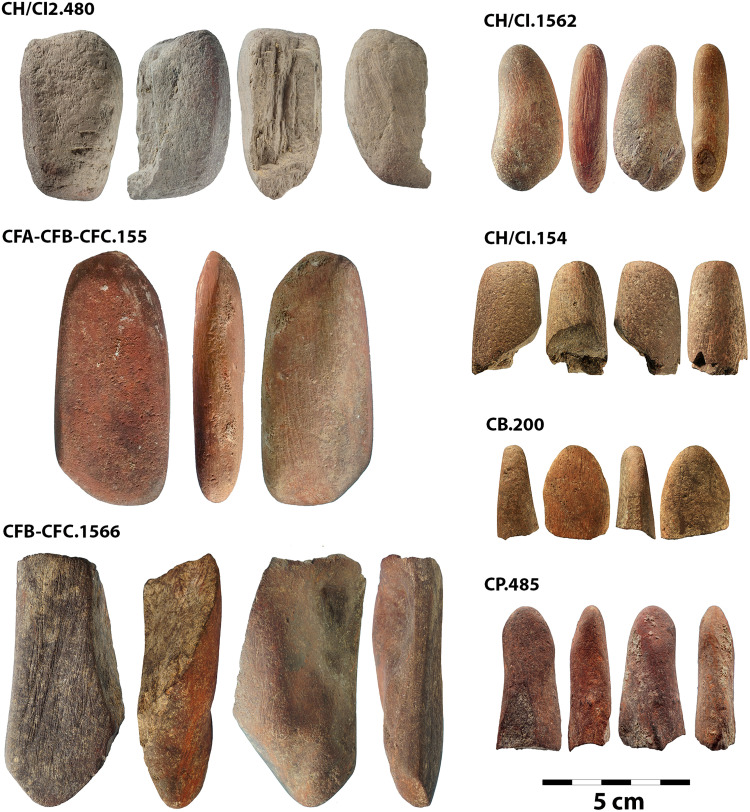
The seven ochre retouchers from the MSA layers of BBC. Artifact numbers are listed in the top left of each separate image.

**Fig. 2.**
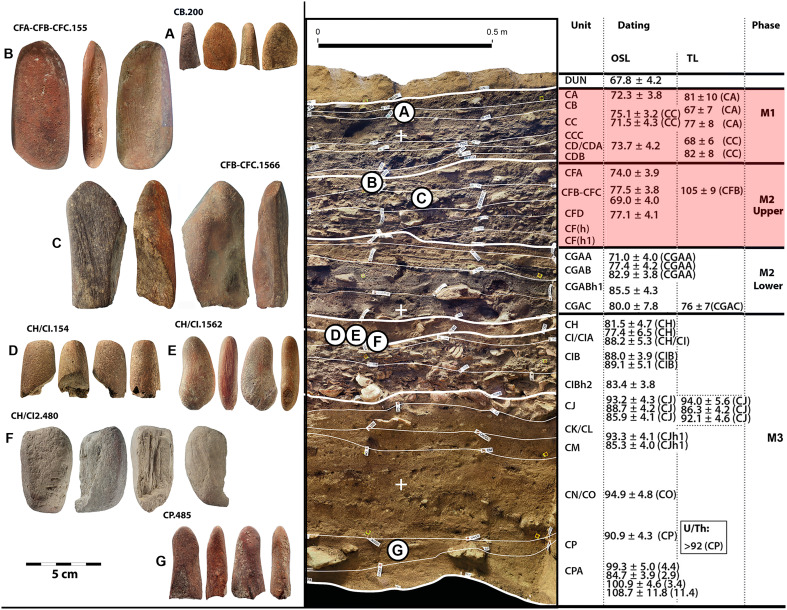
The MSA sequence of BBC (southern section). The figure shows images of the ochre retouchers organized stratigraphically (left), archaeostratigraphic units (middle), and main occupation phases (right). Optically stimulated luminescence (OSL) age estimates from (*21*) and thermoluminescence (TL) ages from (*54*). The Still Bay occupational phases are highlighted in red.

Seven ochre retouchers (Fig. 1 and table S1) were identified from the M3 to M1 phases at BBC (Fig. 2). In the following text, we provide more detailed information on each retoucher chronologically, from youngest to oldest.

#### CB.200

**Fig. 3.**
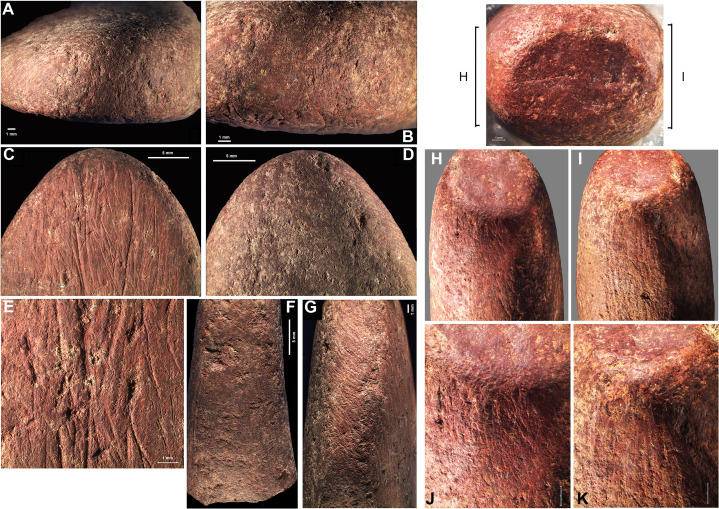
Macroimages of use traces on artifact CB.200 and CH/CI.154. (**A**) and (**B**) notches are consistent with lithic retouching, and (**C**) shows scraping marks on the tip of the piece, overlain with peck marks, with a close-up shown in (**E**). (**D**) shows deep impact scars on the tip with peck marks below. (**F**) and (**G**) show lateral aspects, with (F) showing pecking marks and (G) showing grinding striations on top of pecking marks. (**H**) to (**K**) show different lateral aspects of CH/CI.154, both exemplifying abrasion marks produced by a tool at the edge of the elliptical face and the striations stemming from these points of impact.

The whole artifact is broad and flat and converges to a point (fig. S5). The middle and top are covered by traces of oblique grinding (Fig. 3, D and G, and fig. S5C), and the bottom features a tangential laminar flake scar regularized by pecking (Fig. 3F). The grinding striations appear to be on top of the pecking marks (Fig. 3G), suggesting that the grinding is probably more recent than the pecking and was likely meant to rejuvenate this area of the tool after heavy use. The piece bears two flattened faces (fig. S5, B and D), one with longitudinal irregular striations covered by pecking marks (fig. S5B). On the top left of this aspect, there is a cluster of comet-like impact scars with striations (Fig. 3C). The striations are curved and were likely produced by the tangential contact of the affected material with the ochre piece. It appears that several impacts were produced by striking a single lithic in succession (Fig. 3E). The other flattened face (fig. S5D) is covered by impacts produced by pecking, and fig. S5C shows pecking and grinding marks and the possible remnants of a facet on one edge of the piece (Fig. 3, F and G), which were damaged by the pecking to shape/rejuvenate that top. Last, fig. S5E and Fig. 3 (A and B) show the tip of the piece with one or two deep impact marks, indicating its use not only as a retoucher but also as a hammer to detach blanks.

#### CFB-CFC.1566

This artifact is long and pointed and bears traces of impact on all prominent areas of its convex surface (fig. S6) and microchipping on the tip, which could be traces of damage due to it being used as a pressure flaker. This is furthermore suggested by striations stemming from this area. Close to the tip is a ground, elongated facet, which might have been created to rejuvenate the damaged area (likely from its use as a retoucher). On one side, a long facet running the length of the piece was produced by scraping (fig. S7, C and D). The traces of scraping are clearly visible on the opposite end from the tip but are faded closer to the tip. This is consistent with its possible use as a pressure flaker, during which handling close to the active area of the tool would have smoothed the traces of scraping.

#### CFA-CFB-CFC.155

The artifact is an elongated piece (fig. S8), and one side is completely smoothed and slightly concave, with irregular pecking marks all over it (fig. S8A). This surface is bordered by smoothed, striated edges (fig. S9, E and F). The piece is whole and shows no signs of breakage. One end bears a striated facet, with deep striations consistent with its use as a retoucher (fig. S9C). The other surface shows deep impact scars, also consistent with its use as a tool, with the marks oriented near one end, which may be suggestive that the tool was used by a right-handed person (fig. S9F).

#### CH/CI.154

The artifact bears striations consistent with grinding on each of the lateral sides of the piece (fig. S10, B and D), while numerous pecking marks are visible on the two broader surfaces (figs. S10, A and C, and S11, B and C), suggesting that the object was intentionally shaped using this technique. Striations stem from both ends of the elliptical surface at the flattened and polished tip (Fig. 3, H to K, and fig. S10E), and small flakes still attached at the point of impact can be seen on the broken end of the piece (fig. S10F). Marks of abrasion produced with a tool can be seen at the point of contact between the flat elliptical face at the end and the two lateral aspects and the striations stemming from the two abraded areas (Fig. 3, H and I). The traces of utilization are similar and symmetrical on both aspects of the ochre piece. The object has a unique morphology, and its break patterns suggest that the piece might have once been much longer and possibly used on both sides (fig. S12).

#### CH/CI.1562

This piece is a small, elongated pebble with microflaking and impact damage at both ends (fig. S13). Impact marks extend on both sides of the tip on the thin edges of the pebble and are associated with striations (figs. S13, B and D, and S16, D and F). On one edge, the striations are deep and may have been done by scraping (fig. S13B). Impacts associated with striations are also present on the two flat surfaces of the pebble (figs. S13, A and C, and S14, G and H). The relation between striations and impacts is unclear, but the impact marks are clearly visible.

#### CH/CI2.480

The whole piece is mostly intact and made of a coarse material (fig. S15). At initial glance, one side appears to be anthropologically removed (fig. S15C), although it is likely part of the object’s original morphology, given the lack of evidence for removal and the presence of pecking marks on the surface. One surface contains pecking marks located near the center of the object, which likely indicate the use of pecking for shaping (fig. S15A). Two sides of the object bear scraping striations (fig. S15, B and D), oriented both parallelly and obliquely. One end shows deep, elongated impact marks (fig. S15E), and the other end bears groups of deep peck marks (fig. S15F).

#### CP.485

The piece is elongated in shape, with one end being completely rounded and shaped with rubbing and pecking (fig. S16, A and C). The other end appears broken and contains a possible flake scar, with some light striations and peck marks (fig. S16A). Some of the surfaces are covered by sediment and hide potential use traces (figs. S16C and S17B). There are deep and irregular impact scars on one side (fig. S16B), and the other side shows rubbing near the tip (possible rejuvenation; figs. S16D and S17A). The tip of the piece is pointed and bears some peck marks and some superficial striations (fig. S17E).

Many of the pieces were regularly reshaped and rejuvenated by grinding before accidental breakage and disposal. The shaping activities, such as grinding and scraping, are also closely associated with pigment powder production, and, thus, these actions may have been undertaken with both goals in mind. The use traces indicate that these pieces were carefully constructed and cared for, likely over an extended period. The morphology of the artifacts indicates that the pieces were created and maintained as specialized tools to knap and retouch lithics.

### Experimental results

Using ochre collected from a nearby outcrop (fig. S4), two experimental pieces were created: EX01 (fig. S18) and EX02 (fig. S19). These were used along with two silcrete flakes (fig. S24) to study the impacts of different retouching methods on the morphology of the ochre pieces. We used the ochre pieces in three different experimental scenarios: retouching by pressure flaking (fig. S20A), retouching by percussion (fig. S20, B to D), and pecking (fig. S20E). The results indicate that the three different techniques produce distinct traces on the ochre—these are described in the text below. Further details on the experimental protocol can be found in the Supplementary Materials.

#### Retouching by percussion

This type of retouching, when keeping the ochre vertical, resulted in a pecked facet, from which thin, superficial, discontinuous striations follow (fig. S21, E to H). When keeping the ochre tool subhorizontal, impact marks on the lateral aspect of the ochre piece were produced, with stemming striations that narrow down toward the edge of the object (figs. S19, A and B, and S22, A to D). The depth of the impact marks and the associated striation depth and length are what distinguish these two types of retouch. They differ from pressure flaking in that an impact mark (peck or notch, distinct from striations) is present.

#### Retouching by pressure flaking

This action produced deep, triangular-profile striations starting from the point of contact of the ochre with the lithic, which taper off in depth near the end of the action (fig. S21, A to D). The point of impact marks and striations are uniquely deep, more so than those observed on both forms of retouching. We thus use this feature as a benchmark for identifying pressure flaking on the archeological pieces.

#### Pecking

This produced distinct notches and punctures when the flat surface of the ochre piece was percussed against the pointed end of the silcrete flake (fig. S23).

### Comparisons to archeological ochres

Some inferences can be made when comparing the experimental traces to those present on the ochre retouchers. First, the traces produced by intentional pecking are present on all the retouchers. We interpret this action in two ways: either as a form of shaping the ochre tools, as the notches are often present without accompanying striations; or for another purpose, possibly to increase the grip during use, as pecking marks do not always alter the shape of the object substantially. They are, furthermore, different from impact scars resulting from lithic retouch, as they appear on areas of the ochres that would not be beneficial for retouching (e.g., flat, faceted sides) and are often not accompanied by radiating striations [see figs. S9F and S11 (B and C) for example].

We also observed various forms of percussive markings that form during lithic retouch on all the pieces in this study. Retouching by vertically orienting the ochre tool to the lithic piece was observed on six of the seven ochre pieces, is the most frequent retouch technique, and is the most common use trace after pecking. The pieces include CB.200 (fig. S5E), CFB-CFC.1566 (fig. S7, A and B), CFA-CFB-CFC.155 (figs. S8E and S9B), CH/CI.1562 (fig. S14, A and B), CH/CI2.480 (fig. S15F), and CP.485 (fig. S17, D and E). We observed retouching by keeping the ochre piece subhorizontal (tangential) on three pieces, which are CFB-CFC.1566 (fig. S7B), CH/CI.1562 (fig. S14, F to H), and CH/CI2.480 (fig. S15, A and B).

We observed that the characteristic marks of pressure flaking on the experimental ochre pieces were deep, triangular-shaped striations. We observed similar markings on four pieces: CB.200 (fig. S5, B and E), CFB-CFC.1566 (fig. S7, A and B), CH/CI.154 (Fig. 3, H to K), and CH/CI2.480 (fig. S15E) and, thus, conclude that these pieces were likely used for pressure flaking of lithics at BBC. Table 1 summarizes the results of our interpretations.

**Table 1.** List of ochre retouchers from BBC. The table provides descriptions of use-wear related to retouching and interpretation of the use-wear based on experimental pieces.

Unit	ID	Period	Use-wear	Interpretation
CB	200	Still Bay	Two deep impact scars on the tip along with a cluster of impact scars on the tip (fig. S5E), scraping and grinding striations on the sides and one surface (fig. S5, B and C), one surface only pecking (fig. S5D), flake scars on the side (fig. S5A) and the bottom, and the bottom broken due to usage (fig. S5F).	Pressure flaking
				Retouch—vertical
CFB-CFC	1566	Still Bay	One surface flattened by scraping (fig. S6A), and the other side was natural with some peck marks and shallow striations (fig. S6C). One side flattened by grinding with some shallow impact scars (figs. S6D and S7, C and D), and one facet at worked end (likely rejuvenated) with impact scars on and surrounding (fig. S7B). Several deep impact scars above the facet suggest pressure flaking, followed by rejuvenation of the surface.	Pressure flaking
				Retouch—vertical, tangential
CFA-CFB-CFC	155	Still Bay	Piece is shaped by grinding, smoothing (fig. S8C), and pecking (fig. S8A). Impact marks, flake scar on the tip (figs. S8E and S9B), and deep chips along the edge, followed by shallow striations (fig. S8B). Facet with striations on one end showing rejuvenation after use (fig. S9D).	Retouch—vertical
CH/CI	154	Pre–Still Bay	Piece is shaped by pecking (figs. S10, A and C, and S11, B and C), deep striations stemming from both ends of the elliptical surface at the end suggest pressure flaking (fig. S12, A and B), and small flakes still attached at the point of impact near the broken end (fig. S10F). Grinding to flatten and rejuvenate areas postretouching (fig. S10, B and D).	Pressure flaking
CH/CI	1562	Pre–Still Bay	Deep impact marks and flake scar on the broad tip (fig. S15A) suggest its use for horizontal retouching, while a narrower tip has shallower marks, suggesting this end for vertical retouch (fig. S15B). Some striations on the side for possible rejuvenation and some superficial scraping on the surface (figs. S14 and S15, G and H)	Retouch—vertical, tangential
CH/CI2	480	Pre–Still Bay	Ground facet to make the natural tip more pointed and symmetrical (fig. S16F), and the surface contains numerous and deep impact marks. Light irregularly spaced scraping marks on three surfaces to maintain shape (fig. S16, B and D), and deep and wide impact marks on the anterior surface suggest (fig. S16A) tangential retouch.	Pressure flaking
				Retouch—vertical, tangential
CP	485	Pre–Still Bay	Large flake scar near the broken end of piece, with radiating striations and peck marks surrounding it (fig. S17A). Some peck marks on this surface, which increase near the tip (figs. S17A and S18D), and the opposite surface is lightly covered by sediment (figs. S17C and S18B). Deep and irregular impact scars on one side (fig. S17B), and the other side shows rubbing near the tip (possible rejuvenation; figs. S17D and S18A). Some light impact marks on the tip with superficial striations (fig. S18E).	Retouch—vertical

## DISCUSSION

Retouchers and pressure flakers made of ochre are so far unknown in the Middle and Upper Paleolithic of Europe. Thus, the artifacts presented here represent an original cultural feature of the southern African MSA. Our study here showcases additional evidence of the diverse applications of ochre as different facets within a technical system, where symbolic and functional practices were perpetually interacting rather than being strictly segregated. Furthermore, highly specialized tools do not emerge spontaneously; the utilization of ochre as a tool in retouching predated the Still Bay period. We anticipate that similar traces will be identified at other MSA sites, indicating a specialized technology beginning in the pre–Still Bay and enduring through the Still Bay and, probably, subsequent technocomplexes.

The appearance of intricately worked bifacial points, artifacts associated with social organization and signaling (shell beads), and the collection and manipulation of coloring materials (ochre and pigments) suggest that people during the Still Bay began to reveal and solidify complex behavioral, technological, and cognitive patterns. Previous studies have explored various aspects of these technologies, such as the use of heat treatment for altering lithic materials and personal ornaments (*26*, *28*), complex chaîne opératoire sequences involving pressure flaking (*27*, *36*), the collection, production, and possible wearing arrangements of shell beads (*37*–*40*), the production of formal bone tools (*41*), abstract engravings on ochre (*25*), the possible collection strategies and subsequent uses and applications surrounding ochre and pigment use (*35*, *42*–*44*), and stone tool hafting using compound mixtures (*14*, *45*). However, the retoucher artifacts presented in this paper prompt two aspects previously not identified or discussed during the MSA: the use of pecking techniques for shaping ochre artifacts and the production of special, personalized tools. Personalized tools have been defined as functional implements that exhibit nonutilitarian modifications, idiosyncratic stylistic choices, or manufacturing features that reflect an individual’s or group’s preferences, skill set, or traditions, beyond purely functional or technological constraints (*46*). In the framework of this study, we compliment the above definition of a personalized tool as a functional implement intentionally shaped for a particular use by a specialist who also owned, curated, and eventually maintained it through resharpening and modification over time. In the following sections, we discuss evidence of these behaviors during the pre–Still Bay and Still Bay period.

### Evolution of knapping and shaping technology in the MSA

Shaping technology during the MSA is often associated with stone tool production, as in the case of bifacial points. Shaping technology can also loosely extend to shell bead production (*22*) and to ochre and pigment use, as some ochre pieces bear several heavily ground facets converging into a point and are often described as “ochre crayons” (*15*). The term crayon, however, comes with a caveat, as it can incorrectly be interpreted as an indication of the artifact’s use as a “utensil” to mark a surface when the intended use of these pieces is often unclear. Before this study, ochre crayons provided the only example of the possible shaping of ochre materials during the pre–Still Bay and Still Bay periods.

As we use it here, pecking involves shaping a stone tool or other material using a hammerstone or another rock to strike and remove small fragments without the intention of producing flakes. Although pecking can produce small amounts of pigment, it is not the most effective form of modification for this purpose. It, rather, is a technique that allows for precise modifications of volume. While pecking has previously been documented on other stone artifacts, such as lithics and grindstones (*47*), its use on ochre has only been briefly mentioned as another modification type, and the term is often used interchangeably with “notching” and “pitting” (*48*, *49*). Recent excavations at Panga ya Saidi in Kenya uncovered an ochre crayon shaped by “pecking and polishing,” although the layer the piece was found in dates to ~25 ka calibrated BP (*17*). We identified traces of pecking on all the retouchers described in this study due to their use as tools for lithic retouching. However, several of the ochre retouchers, namely, artifacts CP.485, CH/CI.154, CH/CI.1562, and CB.200, contain distinct pecking marks on the lateral surfaces. In particular, the markings on artifact CH/CI.154 are extensive and cover both lateral surfaces almost entirely (fig. S10). We interpret these markings as indicative that another tool or implement was used to intentionally shape the artifact.

Thus, the artifacts offer evidence of pecking as a direct technique to shape ochre, or, perhaps, with a secondary purpose of pigment procurement, beginning during the pre–Still Bay at BBC (ca. 90 to 92 ka BP) and that persisted into the Still Bay. With a lack of evidence of being used as “crayons” or direct pigment applicants, the pieces offer distinct examples of shaping technology used to create highly specialized tools.

The pieces we interpret as being used for pressure flaking (CB.200, CFB-CFC.1566, CH/CI.154, and CH/CI2.480), except for CH/CI.154, show evidence for also being used in vertical retouching. Artifact CH/CI.154 is unique among the set of retouchers at BBC—modifications on the two sides of the object are identical, as well as the flake scars and striae (Fig. 3 and fig. S10). The tool is shaped as a “barrel” (fig. S12) and was likely abandoned because it broke. When viewing the object vertically, the striations are not perfectly symmetrical on either side (Fig. 3, H and I), and the twisted striae could indicate handedness. We note that it is difficult to find this shape in nature, as it contains a special morphology. Its maker knew the size needed for this specific piece to serve this specific function of pressure flaking. We believe that this specific artifact was not just a piece of ochre used for an expedient task and was likely crafted specifically for being used as a pressure flaker in specific phases of the tool shaping reduction sequence. The user (and possibly maker) was a specialized knapper and already knew how to create an object tailored to pressure flaking in the pre–Still Bay.

### Personalized ochre tools and behavioral implications

The patterns of use and rejuvenation of some of the tools, namely, CFB-CFC.1566, CFA-CFB-CFC.155, and CH/CI.154, suggest that they were specifically created, curated, and transported by a craftsperson for knapping and, perhaps, indicate that these were personal belongings, or crafted for and by a specific person or rather different individuals with a common form in mind, likely stemming from a shared cultural or social tradition (*46*). The ochre used for shaping this object is exceptionally hard, which is a key factor in its suitability for this function. While we selected our experimental pieces for their high hardness, they did not perfectly match the properties of the archeological specimen, which may have influenced the degree of efficiency observed in our tests. While bone is a well-documented material for pressure flaking, the use of ochre suggests that factors beyond pure functionality may have played a role. Given that ochre was also used in symbolic activities, it is possible that these objects carried a special meaning, potentially as personal- or status-related items. Their use in tool production may not have been purely pragmatic but could reflect aspects of cultural identity, ownership, or even ritual significance. Our findings may indicate the emergence of individual property in human societies from a very early date, or at least the recognition of certain tools crafted by or for specific people for defined practices. The elongated shape of several pieces (CFA-CFB-CFC.155, CFB-CFC.1566, CH/CI.154, and CP.485) suggests a special activity requiring a certain level of expertise. Their specific shape denotes their creation as specialized tools, with a modified morphology, made for a certain activity. Their elongated shape makes it easier to damage the point that is actively in contact and indicates a certain skill level, which must have been learned and mastered over time, to properly use them as effective tools. These skills and techniques were likely passed down through generations. Given the temporal presence of these tools, it is likely that they were originally more numerous than the archeological remains suggest.

The pieces also offer a unique perspective into the cognitive capacities of pre–Still Bay peoples inhabiting BBC. Specifically, the pieces show an ability to impose a preconceived geometric form executed through pecking and, in some cases, grinding and scraping. These actions, while used to shape some objects, may also have concurrently resulted in producing ochre pigment powder, especially in the case of grinding and scraping. At this point, it is difficult to identify whether the actions were executed exclusively to shape the objects and powder production was a beneficial by-product, or vice versa. They nonetheless indicate a knowledge and comprehension of the outcomes when creating these ochre tools. This observation further deconstructs the interpretive duality of ochre into “formal” and symbolic categories and offers further evidence that ochre behaviors likely operated on a multitude of realms simultaneously.

Artifact CH/CI.154, before breakage, exhibited an almost perfectly flattened bitruncated ellipsoid shape (fig. S12). To date, no similar pieces exist in Africa or Europe before the European Upper Paleolithic (*50*, *51*), especially ones shaped using similar techniques. The earliest examples that resemble this shape and form are stone beads in the Neolithic Levant (*52*). Conceiving and producing such a specific form represents a cognitively and technologically challenging endeavor and is not a skill previously attributed to the pre–Still Bay.

When looking at the temporal association of the ochre retouchers, more than half come from the M3 layers (CP, CH/CI, and CH/CI2). Three of the pieces are from the upper M3, which Watts (*35*) reports as the most ochre-rich of all of the layers at BBC (particularly layers CJ to CH). In these upper M3 layers, there are also at least 10 engraved ochre pieces (*25*). However, engraved ochres are reported from the entire sequence and exemplify a technological/symbolic tradition that persisted over millennia at the site. The engraving of ochre, coupled with its use as a specialized tool and the creation of such specialized tools out of ochre, highlights a level of symbolic expression coupled with a technological perception that suggests a nuanced understanding and utilization of ochre beyond mere functional purposes. In layer CP, two ochre processing toolkits and evidence for ochre mixtures and compounds (*20*) offer further evidence that ochre was used and applied as a “multi-use tool” during the M3 at BBC.

In conclusion, the lithic and ochre technologies at BBC reveal a dynamic interplay between symbolic and functional practices. The presence of specialized ochre tools for retouching, specifically pressure flaking, predating the Still Bay period suggests an emergence of an innovative technology and its development over time. The appearance of finely crafted bifacial points, shell beads indicative of advanced social organization, and the manipulation of ochre and pigments to create geometric designs (*24*, *25*) or complex paint mixtures (*20*) underscore complex behavioral, symbolic, and cognitive patterns. Our findings on the utilization of pecking techniques to shape ochre artifacts, evidence for pressure flakers during the pre–Still Bay and evidence for the production of personalized tools, shed light on early concepts of property and specialized craftsmanship. To date, no evidence has been found for pressure-flaked lithics during the pre–Still Bay, although only a small subsample has been studied in depth (*53*). Thus, it is possible that, in future studies, such traces will be uncovered. Regardless, we argue that pressure flaking technology was conceptualized in the pre–Still Bay and further developed into the Still Bay. Furthermore, in the same study, Douze *et al.* (*53*) note that some 25% of informal tools from Marine Isotope Stage 5 bear ochre residues. The assemblage from BBC stands out as compelling evidence of ochre’s utilization for both symbolic and functional purposes concurrently. As we continue to explore its capabilities and cultural significance throughout the African MSA, we can unravel the profound ways in which ochre enriched the lives of our human ancestors.

## MATERIALS AND METHODS

General artifact photos were taken with a Canon PowerShot 67× and a Sony α6500 digital camera with a 3.5/30 macro lens. Microscopic photos were taken with a motorized Leica M125 equipped with PLANAPO objective, a DFC295 digital camera, and a Leica Application Suite v.4.5 Software (Leica, Wetzlar), including the multifocus module. The multifocus module combines multiple digital images taken at different *Z* positions into one single sharp, fully on-focus composite image.

For the experiments, we used two pieces of ochre collected near the town of Suurbraak, some 60 km northwest of BBC (fig. S4). We selected this material for experiments as it is homogeneous and similar in texture and color to most of the ochre pieces bearing possible use traces produced by retouching activities. From this material, we created two experimental pieces (EXR01 and EXR02), replicating the shape and size of one of the retouchers (CH/CI.154). On the replicated ochre pieces, we used two silcrete flakes (fig. S25) recovered from reworked sediments at BBC to test different methods and techniques of lithic retouching and ochre pecking (and any other associated marking and use traces). A more detailed overview of the experimental design can be found in the Supplementary Materials.

## Supplementary Materials

### This PDF file includes:

Supplementary Text Figs. S1 to S24 Table S1 References
