# Supplementary material for: Unveiling the multifunctional use of ochre in the Middle Stone Age: Specialized ochre retouchers from Blombos Cave
Source: Sci Adv. 2025 Jun 27;11(26):eads2797. doi: 10.1126/sciadv.ads2797 (PMC12204176; doi:10.1126/sciadv.ads2797)
Supplement: Supplementary file 1 — Supplementary Text Figs. S1 to S24 Table S1 References [file sciadv.ads2797_sm.pdf]

Science Advances
AAAS

Supplementary Materials for

Unveiling the multifunctional use of ochre in the Middle Stone Age: Specialized ochre retouchers from Blombos Cave

Elizabeth C. Velliky *et al.*

Corresponding author: Elizabeth C. Velliky, elizabeth.velliky@uib.no

Sci. Adv. **11**, eads2797 (2025)
DOI: 10.1126/sciadv.ads2797

This PDF file includes:

Supplementary Text
Figs. S1 to S24
Table S1
References

1. Archaeological Background

Located in Blombos Private Nature Reserve some 300 km east of Cape Town, Blombos Cave (BBC) stands 34.5 m above modern sea level and about 80 m from the present-day shoreline along the southern coast of the Western Cape province of South Africa (fig. S1). It is located within a south-facing calcarenite cliff in the Table Mountain Group (TMG) sandstones (folded and faulted Paleozoic sediments of the Cape Supergroup) and overlain by the Cenozoic formations of the Bredasdorp group (*56*). In front of the cave mouth and dripline there is a gently sloping talus platform of about 25 m2 (fig. S2) which extends, then sharply drops, some 34.5 m southward toward the shoreline below. The talus is stabilized by several large rocks and blocks, which have allowed both Later and Middle Stone Age materials and sediments to accumulate and remain protected from weathering and erosion (*54*).

Excavations began at Blombos Cave in 1991 and, with some pauses in between, were actively ongoing until 2024. The cave contains both Later Stone Age (LSA) and Middle Stone Age (MSA) contexts, and the LSA layers were originally capped with about 20 cm of sterile eolian deposits. The current excavation area consists of one unit measuring ca. 19 m^2 in size within a ca. 41 m^2 sized cave interior (behind the dripline). Most of the anthropogenic layers that have been excavated come from this area.

1.1. The Archaeological Assemblage

1.2. The lithic sequence

The M1 and Upper M2 phases (71-76 ka) contain lithics that have been classified as belonging to the Still Bay (SB) technocomplex due to the presence of over 500 bifacially worked stone points. These points typically have a narrow elliptic to lanceolate shape and two sharp apices (fig. S3) (*29, 57*) The majority of SB points were made using coarse and fine-grained silcrete (>70%), while some were made using quartzite (c. 15%) and quartz (c. 13%). At BBC, Villa et al. (*26*) identified four main production phases for SB points, including hard hammer and direct percussion in the initial reduction phase (phase 1), followed by soft hammer and marginal percussion (phase 2), pressure flaking during the final retouch phase (phase 3), and a few points that were reworked by hard hammer percussion (phase 4). A previous study by Mourre et al. (*27*) based on experimentally replicating some bifacial points, showed that many of the silcrete points were likely heat-treated before being finished using pressure flaking. Many of the bifacial point production rejects (89.8%) suggest that point production was a primary, though not exclusive, activity at the site. Some evidence suggests that at least some of the bifacial points were used as spear tips, with indications of axial hafting (*27, 58, 59*). Therefore, it is likely that most of the finished points were removed from the site and used for hunting or as multi-functional tools elsewhere.

Regarding the general lithic assemblage, Henshilwood et al. (*57)* conducted a preliminary analysis of around 60,000 lithic fragments, while Douze et al. (*53*) provided a techno-cultural characterization of 3,404 lithics (>2 cm) from the M3 levels, making it the most comprehensive documentation of the MSA lithic assemblage found at BBC. The lithic assemblage contains various formal tool types,

including flakes, blades, bifacial and unifacial points, scrapers (side, end, circular), notched pieces, burins, borers, and denticulates. The abundance of formal tools changes throughout the MSA sequence. The M1 phase is dominated by silcrete (>70%), followed by quartz (16%) and quartzite (14%). The upper M2 phase has more quartz (>50%) and less silcrete (30%). The M3 phase is mainly dominated by silcrete (62%), followed by quartzite (19%) and quartz (17%).

In contrast to the M1 and Upper M2 phases, the M3 phase is characterized by the production of blanks, including different types of blades and triangular flakes, primarily through extensive in-situ core reduction. Retouched tools are rare in the M3 phase. Douze et al. (*53*) concluded that the overall technological organization of the M3 assemblage should be assigned to the stable, blank-producing techno-cultural tradition, which was widespread on the southern Cape coast between 105 and 90 ka ago (MIS 5c to 5b).

1.3 Symbolic material culture

The discovery, almost two decades ago, that marine shells belonging to the species *Nassarius kraussianus* were used as beads at Blombos Cave, in layers dated to ca. 73 ka (*22, 40, 60*) was the first to challenge the longstanding view that ornaments were first used in Europe at c. 40 ka. Taphonomic analyses and experimental reproduction of the modifications recorded on the shells have shown that *N. kraussianus* from Blombos Cave were collected alive and perforated through the aperture with the help of pointed tools, probably bone awls found in the same layers. Experimental data and chemical analyses indicate that the shells were probably heated in a reductive environment, to change their colour (*38*). Experimental reproduction of use-wear recorded on the archeological beads has indicated that a change in the way beads were strung and the visual appearance of the resulting beadwork occurred at this site between lower and upper Still Bay layers (*22*).

A recent study has revealed that between 100 and 73 ka unperforated and naturally perforated eye-catching shells of *Semicassis zeylanica*, *Conus tinianus* and another unidentified *Conus* species were brought to the cave. At ca. 70 ka a new species of marine gastropod, *Triviella ovulata,* was intentionally perforated and worn as an ornamental object, either on its own or with other intentionally perforated *N. kraussianus beads.*

In addition to the shell beads, approximately 40 bone tools have been recovered from the MSA levels, with awls being the most prevalent tool type (n = 25) (*41, 61*). These awls were crafted from long-bone shaft fragments and meticulously shaped through extensive scraping and were likely utilized for piercing soft materials like animal skins or shell beads. Previous studies on similar bone tools (*62*) have proposed that bone awls could have been linked to the technology needed for processing animal hides, and are also perhaps evidence for clothing manufacture and tailoring. In addition, six polished MSA bone points, which may have been used as hafted projectile points have been discovered, and one bone tool shows signs of having been used for lithic retouching (*41*).

Additionally, a modified bone fragment with eight parallel lines was recovered from the Still Bay layers (*63*) while a long-bone shaft fragment exhibiting a sequence of oblique incisions was found from the M3 phase. According to d'Errico and Henshilwood (*61*) these incisions were intentionally and carefully executed and do not resemble typical butchering marks.

1.4 The BBC ochre assemblage

Anthropogenically modified and non-modified iron-rich earth materials, colloquially referred to as ochre, have been recovered throughout the MSA sequence at BBC. The ochre assemblage was originally reported in 2001 (*57*) and a detailed analysis followed in 2009 by Ian Watts (*35*). In Watts' initial assessment, he reports some 8,224 pieces from the 1998/1999 excavations at the site, weighing a total of 5831g. Of these, he reports some 1,448 pieces measuring over 10 mm, with the majority (79.1% by number and 82.4% by mass) coming from the M3 layers (formerly labeled as BBC 3) at the site. He reports that most of the anthropogenically modified ochres (n = 1,534) showed deep and saturated shades of red, and typically exhibited striated surfaces from intensive grinding, indicating pigment (powder) extraction. While the majority of currently reported modified ochre is associated with the upper M3 occupation levels, a substantial amount (n = 254) has also been found in the Still Bay phases. An analysis of the BBC ochre assemblage from the excavation seasons following 1999 is currently underway.

In 2002, two modified ochre pieces, both from the M1 phase, were published and feature cross-hatched scorings in combination with parallel incised lines (*23*). Another 13 pieces were reported from the entire sequence in 2009 (*25*). Another highly modified ochre piece, also with parallel incised lines, was recovered from the upper M2 phase, gathered in-situ in an impregnated block section and reconstructed using micro computed tomography (*64*). Henshilwood et al. (*25*) suggest that the geometric arrangement of the incisions (or score marks) on these and similar ochre pieces are engravings that may represent a premeditated pattern or design. The intentionally engraved pieces appear to have been subjected to highly consistent scoring pressure indicating that the lines were produced with precision and control. The surfaces bearing the engravings are sometimes prepared by intensive grinding in order to create a flat surface, indicating a certain level of cognition due to the intricate, pre-meditated steps of planning and execution and thus may be indicative of early symbolic behaviour (*25, 65, 66*).

Aside from the physical ochre pieces, other artefacts associated with ochre include a silcrete flake displaying a cross-hatched pattern drawn on it with an ochre crayon from the Still Bay (*24*). The designs on this artefact bear similarities to the engraved ochre pieces and not only demonstrates that drawing was practiced by the Still Bay inhabitants at BBC, but also "… their ability to apply similar graphic designs on various media using different techniques," (*24*). Additionally, an in-situ toolkit consisting of ground ochre powder, heated seal bone, and charcoal mixed into a liquid compound and stored in two abalone shell containers from the cave's lowest level, dating back to approximately 101 ka was reported in 2011 (*20*).

1.5 Discovery of the ochre retouchers

During the recent qualitative documentation and cataloguing of the Blombos Cave ochre assemblage, several anthropogenically modified pieces were put aside for further investigation. The unique shaping and pecking marks observed on the retoucher pieces were immediately noted as unique and not consistent with other types of ochre modification that are indicative of pigment powder production through grinding, rubbing, or pulverizing. Upon further microscopic examination and thorough assessment of the characteristic lateral pecking and percussion marks on the tips with striae radiating out from these, it was determined that the artefacts represented a new type of ochre "tool" that had previously not been documented at a South African MSA site or elsewhere. Some "peckings" resembled those known on Middle Palaeolithic bone retouchers from Europe and Asia (*67-70*) and previously recorded on a shaft fragment from Blombos Cave M3 phase, layer CJ (*61*). Other markings, associating microchipping and striations shared similarities with bone and antler pressure flakers reproduced experimentally and identified archaeologically at many prehistoric sites around the world (see *71, 72-75*), including in the Middle and Early Later Stone Age layers of Sibudu (*34*).

2. Analysis of archaeological and experimental ochres

2.1. *Macro- and microscopic investigation*

The ochre assemblage at BBC was first macro- and microscopically examined and documented in the SapienCE Satellite laboratory in Cape Town, South Africa, where the majority of the BBC collections are housed. The documentation involved common descriptive techniques used in MSA ochre studies (*35, 48, 49, 76-78*), including stratigraphic provenience, size (length, width, depth in mm), weight (in g), colour, texture, type (friable shale, indurated shale, sandstone, ferricrete, etc.), flake scars, and any other visible anthropogenic use-traces, including striations, score marks, facets, pecking, shaping, and the various intensities of these. The assessment of the ochre assemblage from the post-1999 excavation season is currently underway, and has revealed an additional 1537 pieces <10 mm.

The thorough assessment, description, and documentation of the artefact assemblage allowed for the characteristic features to be noted on other modified pieces. In total, seven ochre pieces possibly used as retouchers were identified from the BBC sequence, spanning from layers CP-CB (table S1). These are described in detail in the main text.

2.2. *Experimental replication of the ochre retouchers*

For the experiments, we used a large chunk of ochre collected near the town of Suurbraak, some 60 km northwest of Blombos Cave (fig. S4)

All experiments were conducted by one of us (FDE). The first piece, labelled EXR01 (fig. S18) was shaped with an ESCIL 300 GTL lapping and polishing machine using a 300-micron carborundum powder to reproduce the size and shape of CH/CI.154 (fig. S10). We first shaped the piece to reproduce the traces on an object of a similar morphology (in this case a truncated ellipsoid). There are three reasons for shaping the object beforehand; firstly, it allows it to be reshaped after each use and experiments can be repeatedly conducted to check the consistency of the results.

Secondly, all traces produced by the tool clearly stand out on a homogeneous, finely ground surface. Lastly, using a symmetrical object (like the archeological one) allows one to conduct the same types of experiments on all sides of the object.

Another experimental piece, named EXR02 (fig. S19) was shaped with the same polishing machine to create a flat surface with two slightly rounded ends, imitating the morphology of CB.200, which is rounded and slightly pointed with a flattened top bearing use-traces (fig. S5).

Figure S18 shows EXR01 prior to use. The objects maintained the same morphology and size after each reshaping with the polishing machine as this only removed ca. 100-200 microns of material from the surface, enough to erase previous traces of utilization. EXR01 was cleaned under running water and dried after each experimental use. We observed that this was the only way to remove the ochre powder deposits adhering to the surface and make the traces comparable to the archaeological traces. As with EXR01, EXR02 was reshaped after each use, cleaned under running water, and dried before being photographed.

We experimentally used EXR01 for pressure flaking (fig. S20A) as well as to retouch a flake edge by percussion following a direction parallel to the object main axis by keeping the object vertical (fig. S20B). We also retouched a flake edge by percussion with the main axis of EXR01 kept perpendicular, or slightly angled, to the direction of the movement (fig. S20C). These techniques were replicated three times. EXR02 was used as a retoucher (fig. S20D) by gently percussing its surface on the pointed end of a silcrete flake or, vice versa, to peck the flat surface of the ochre with the flake tip (fig. S20E).

Figure S21 shows two examples of the use of EXR01 for pressure flaking (fig. S21A-D) and as a retoucher by percussion keeping the object vertical (fig. S21E-H). Figure S22 shows two examples of EXR01 being used as a retoucher while kept horizontal during the retouching process. Figure S19 shows the traces produced with the same motion on EXR02. Figure S23 shows the result of pecking on EXR02.

We recognize that comparisons between experimental and archaeological specimens are subject to inherent challenges. While we attempted to conduct the experiments in a consistent and controlled manner, several factors may have influenced the results. Differences in the physical and mechanical characteristics of the experimental raw materials—such as the precise hardness, grain structure, and brittleness of the ochre—may have affected the replication of observed archaeological patterns. The manual dexterity, experience, and cognitive approach of modern experimenters likely differ from those of prehistoric toolmakers, who may have refined these techniques over extended periods of practice and cultural transmission. This gap could influence the accuracy of our experimental replications. As is well documented in experimental archaeology, similar traces can result from different processes, making it challenging to unambiguously correlate our experimental observations with specific past behaviors. Finally, while experimental archaeology is a powerful tool for testing hypotheses about past behaviours, it does not guarantee that all potential techniques and processes used by prehistoric artisans are replicated in modern experiments. Despite these limitations, we believe that our experimental approach provides

valuable insights into the potential mechanics of ochre processing and stone tool modification.

3. Results

3.1. *Experimental results*

The experimental results show that the use of EXR01 in the three different retouching techniques produces distinct use-traces. Using the replicated piece as a pressure flaker resulted in deep distinct striations starting from the point of contact of EXR01 with the lithic. Using the piece as a retouching tool, oriented vertically, resulted in a pecked facet from which thin, superficial, discontinuous striations radiate outwards. The use as a retoucher by percussion, keeping the object sub-horizontal, produced impacts on the lateral aspect of the experimental piece, as well as striations that narrow down toward the end of the object. Similar traces are observed on EXR02 when used in the same way and, with variations, to many of the archeological retouchers found at BBC. When compared with the traces on some of the archaeological retouchers (e.g., CH/CI.154) we believe that, although eroded, the deep striations produced by pressure flaking are still visible and the pecked surface associated with the vertical percussion is absent, suggesting pressure flaking is the practice more parsimonious with the use-traces present.

Both percussive and pressure retouching were effective in producing microflake scars on silcrete flakes. However, pressure flaking did not result in the invasive removals observed on Still Bay points. This is likely due to several factors: (1) Still Bay preforms were heat-treated to enhance isotropy (*6*), (2) the platforms where pressure was applied were carefully prepared, and (3) the silcrete used in our experiments had a coarser texture, which may have affected flaking efficiency.

4. Discussion and Conclusion

4.1. *Still Bay lithic technology*

The main identifying feature of the currently known Still Bay sites is the presence of bifacial foliate points, which are typically made from fine grained raw materials (*26, 57, 79*). Previously, it was thought that Still Bay bifacial points were only found in the coastal region of the Western Cape Province, but discoveries at two sites in KwaZulu-Natal, Sibudu (*80, 81*) and Umhlatuzana (*31)*, and at Apollo 11 in Namibia (*82*) have disproved this notion. The production process involves bifacial roughouts at various stages, with flakes generated during thinning and shaping regularly recovered at Still Bay sites. At Blombos Cave, most bifacial points were made on heat-treated silcrete to facilitate pressure flaking and retouch (*27*) and served as versatile tools. They were used as both spear points for hunting and knives (*27, 58*).

Outside of technological functions, the Still Bay bifacials may have held a symbolic significance (*83*). The high level of technical skill required to manufacture Still Bay bifacials suggests that the people who created them were likely to have had a culture that was mediated by symbols, a trademark often tied to the emergence of "modern human behaviour" (*10, 84*). Henshilwood and Dubreuil (*85*) define a symbolically

mediated culture as one in which individuals understand that artefacts are imbued with meaning and that these meanings are constructed through collectively shared beliefs. The use of non-local raw materials and the complex manufacturing process of Still Bay bifacials, such as heating the materials and using various flaking techniques (including pressure flaking), suggests that they may have had a value beyond their functionality. This could have included adding exchange value to the tools and promoting social relations (*86, 87*).

While pressure flaking allowed for thinner points to be produced, which may have had greater penetrative power than thicker, blunter points, the sophistication of the manufacturing process and the use of high-quality raw materials suggest that other factors, such as social value, may have also played a role in the creation of these tools.

4.2. *Ochre modifications and behavioural implications*

One of the main observations outlined in this study is the use of pecking to intentionally shape pieces of ochre. Pecking (also referred to as "notching" or "pitting") is a form of anthropogenic modification found on ochre artefacts during the MSA (*15, 48, 49*), though it is not as frequently reported as striations from grinding, flaking, polish, or rubbing. Prior to this study, the earliest report on ochre pecking comes from Pinnacle Point 13B from layer DBS 3, dated to 91–102 ka BP (*49*). Here, Watts reports a chunk of fine-grained sandstone that was intensively ground in addition to being scraped and notched. The piece itself is < 5 cm, and the notching does not appear to have been applied with the deliberate intention of shaping the artefact (p. 406, fig. S7D). In addition to this piece, he reports on other notched ochre artefacts that came from profile cleanings and thus have no provenance, though he attributes these as likely originating from layers DBS 3 - LBS 1 (ca. 91 – 94 ky BP) (*88*). He reports on one of the pieces: "No use-wear was observed in the notches, but on one face grooves extend from both notches, almost meeting in the center. A speculative interpretation... is that the notches, together with the grooves, aided suspension of the piece," (p. 406). Of the other piece, which measured ca. 5 mm and likely broke off a larger piece, he notes one edge with two distinct notches that likely served a different purpose. He furthermore notes that these conclusions need to be tested with more intensive investigation and microscopy. Additionally, he did not suggest that notching or pecking was performed with the direct intention of producing pigment powder. Previous ochre experiments on producing pigment powder do not include pecking (*89*), and other studies which identify pecking or "pitting" on archaeological ochres associate the activity with attempts to break the pieces to produce smaller flakes (*48*).

At BBC, edge "notching" was noted on one of the engraved pieces (p. 37, fig. S18) reported by Henshilwood et al. (*25*). The artefact, which is two refitted pieces of siltstone, is from the same layer as the heavily pecked piece CH/CI.154. However, here we note that the visual classification of "notching" appears to denote a different type of feature than "pecking", as peck-marks are observable on several of the other engraved pieces from BBC. This further supports the arguments discussed in Haaland et al. (*64*) who outline the many discrepancies in ochre modification

terminology, how these terms are often reflective of the behaviour and not necessarily features of the modification and finally argue in support of "…the need and the benefits of quantitatively characterizing and classifying ochre surface modifications in a standardized and consistent way."

The presence of intentionally shaped ochre tools at BBC throughout the sequence showcases an innovative ochre technology which arose in the M3 and was passed down over time into the Still Bay. The earlier pieces are naturally shaped, but prior to the onset of the Still Bay, suitable and perhaps desirable ochre pieces were selected and intentionally shaped with this technological aspect in mind, beginning with CH/CI.154. Although the actions producing the specific tool shape could have simultaneously produced pigment powder, we do not interpret this as the primary goal of the tools' creators given the high number of other types of use-wear less consistent with pigment production (pecking, percussion marks). Thus, this behaviour exemplifies a unique and specialized technological adaptation by the inhabitants of BBC.

Given the lower frequency of ochre retouchers throughout the sequence, and the availability of other, perhaps more readily available materials like stone or bone, it is difficult to speculate the drivers behind their creation and use. Though we have shown ochre to be an effective retoucher and pressure flaker, further experiments could establish whether these perform better than other osseous or lithic materials as a hard or soft hammer. Furthermore, ochre is not a scare resource on the southern Cape and is readily available some >15 km from the site in the extensive Bokkeveld Shale deposits. These outcrops were likely accessed regularly by the BBC inhabitants, given the frequency of ochre at the site and of iron-rich shale in the assemblage (*35*). Thus, ochre was as available and accessible as bone or lithic raw materials, which suggests that other interpretations may account for its use as a lithic retoucher.

In addition to lithic retouching and Still Bay points, it is possible that the pieces might have been created as particular tools that have specific advantageous attributes as knapping instruments. Perhaps the use of specialized ochre retouchers, or the specialized synergy or combination within the reduction sequence offered further advantages that we currently do not see. There is also the possibility of a symbolic characteristic to the ochre tools, whether they fulfilled a certain ritual or ceremonial aspect to the retouching of lithics. As d'Errico (*90*) suggests, ""There is no traditional society in which the production and use of colourant is merely functional." It is not such a far stretch to extend this observation to physical ochre artefacts and the actions that they can perform, thus it is possible that these tools operated in several spheres of both symbolic and functional behaviours.

4.3 Conclusion

The diverse occupational patterns observed during the M3-M1 phases at BBC offer valuable insights into the technological evolution and cultural dynamics of Middle Stone Age (MSA) populations. The lower M3, characterized by a general scarcity of materials and high raw material variability, suggest short-term occupations with imported lithic elements, possibly indicating targeted activities during transient stays

(*53*). Layer CP, with its ochre processing toolkits, provides a good example of such focused activities. The upper M3 dominated by silcrete and dense shellfish assemblages, indicate longer-term occupations with *in-situ* knapping activities and domestic tasks, reflecting a shift in occupational patterns. While there is general lithic technological stability during the M3, ochre-related behaviours not only emerge but diversify and flourish during the M3 and persist into the M2 and M1 phases. The creation of ochre mixtures for paint (*20*), ochre engravings (*25*), ochre drawings on silcrete flakes (*24*), and the crafting of ochre tools for specific technological purposes (as shown in this study) signify a rich ochre-based technological tradition at BBC, suggesting concurrent symbolic and functional practices. These discoveries offer insights into the cognitive capacities of pre-Still Bay populations, which challenge previously described timelines of technology and argue for a broader understanding of human innovation during the Middle Stone Age.

SM Figures and Tables

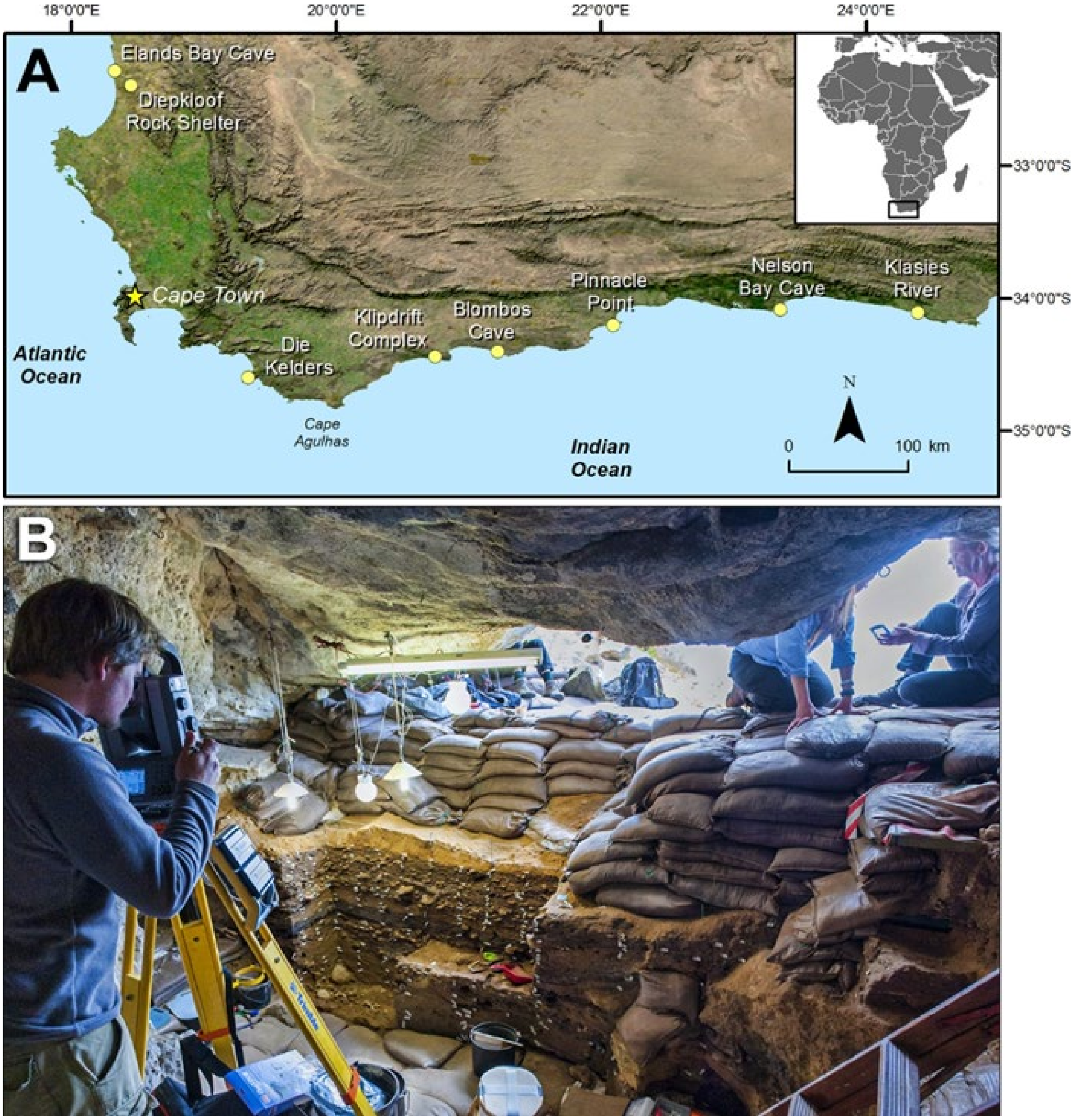

Figure S1: Location of Blombos Cave, South Africa. Detail A shows the site in relation to other key MSA sites and (B) shows a photograph of Blombos Cave interior, toward the cave mouth. Photo credit for detail B: Magnus M. Haaland, University of Stavanger. Original publication (*55*), used with permission (CC BY 4.0).

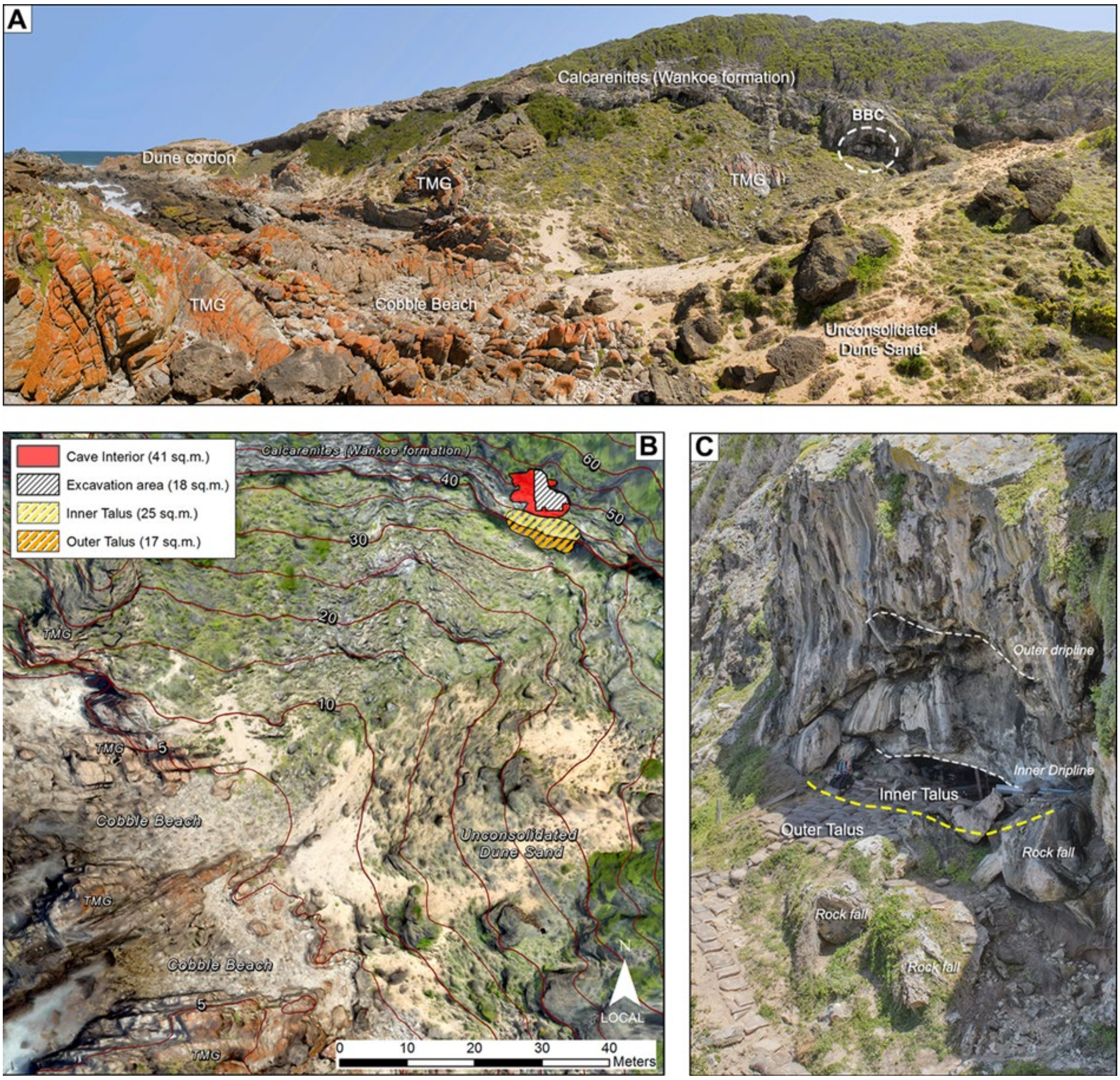

Figure S2: Blombos Cave landscape setting and context. Detail (A) shows landscape and topographic features outside Blombos Cave (BBC); (B) planar overview of Blombos Cave and its surroundings; and (C) close-up view of the Blombos Cave talus and entrance area. TMG, Table Mountain Group. (Image by M.M. Haaland). Photo credit for all details: Magnus M. Haaland, University of Stavanger. Original publication (*55*), used with permission (CC BY 4.0).

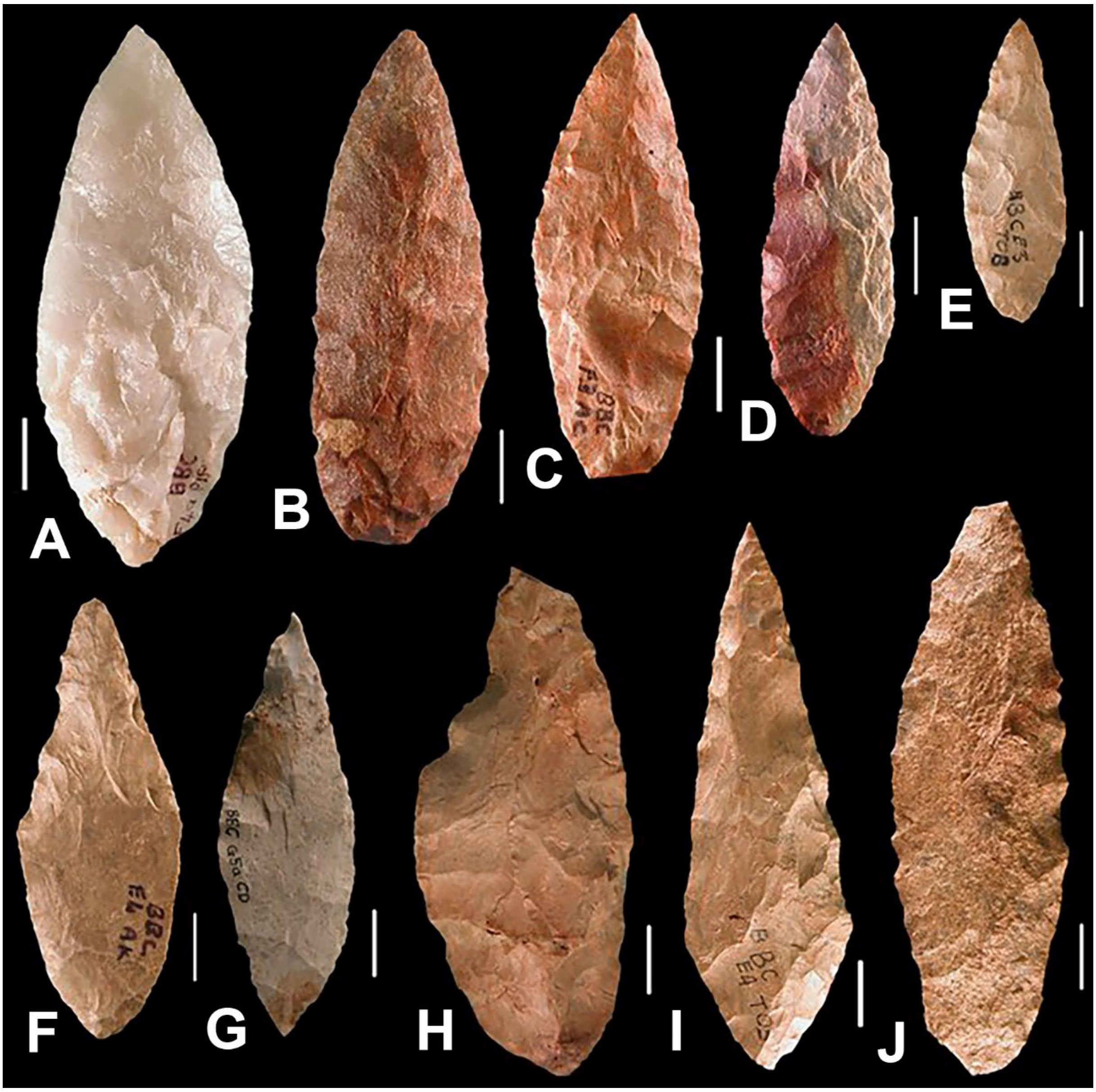

Figure S3: Examples of Blombos Cave Still Bay bifacial points. All made of silcrete except no. 1 of quartz. (A) PVN 63 E5b BZ; (B) PVN 7 D4b CD; (C) Museum3 F3 AC; (D) P54 E6a CC; (E) Museum1 E3 TOB; (F) PVN 65 E4 AK; (G) P 71 G5a CD; (H) PVN 140 E3 TOB; (I) PVN 68 E4 TOB; (J) PVN 71 E4 PIP. Scale bars = 1 cm. Original publication (*29*), used with permission (CC BY 4.0).

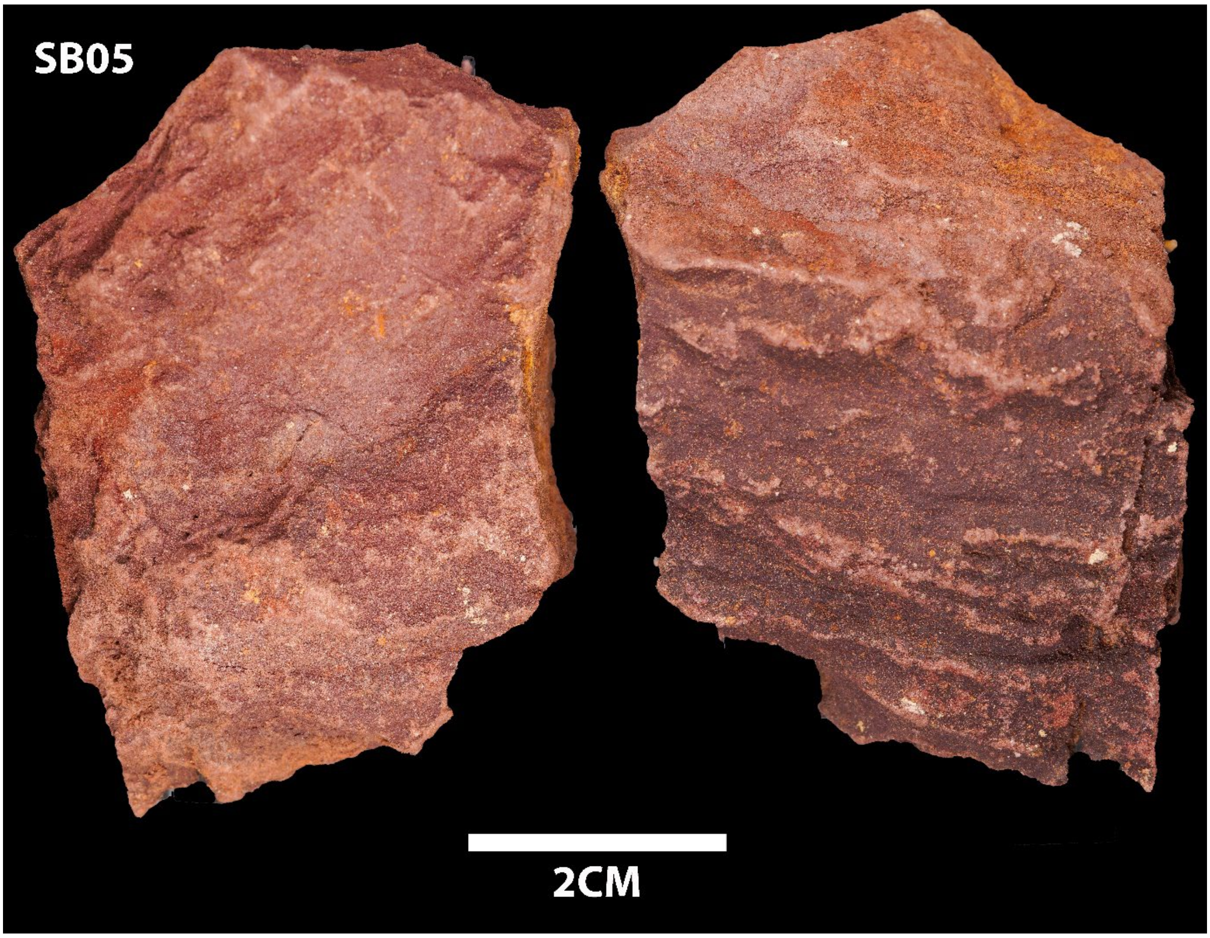

Figure S4: Ochre used for experimental retouching. The ochre was collected from outside of Suurbraak and used for replicating the experimental retouchers used in this study. Photo by E. Velliky.

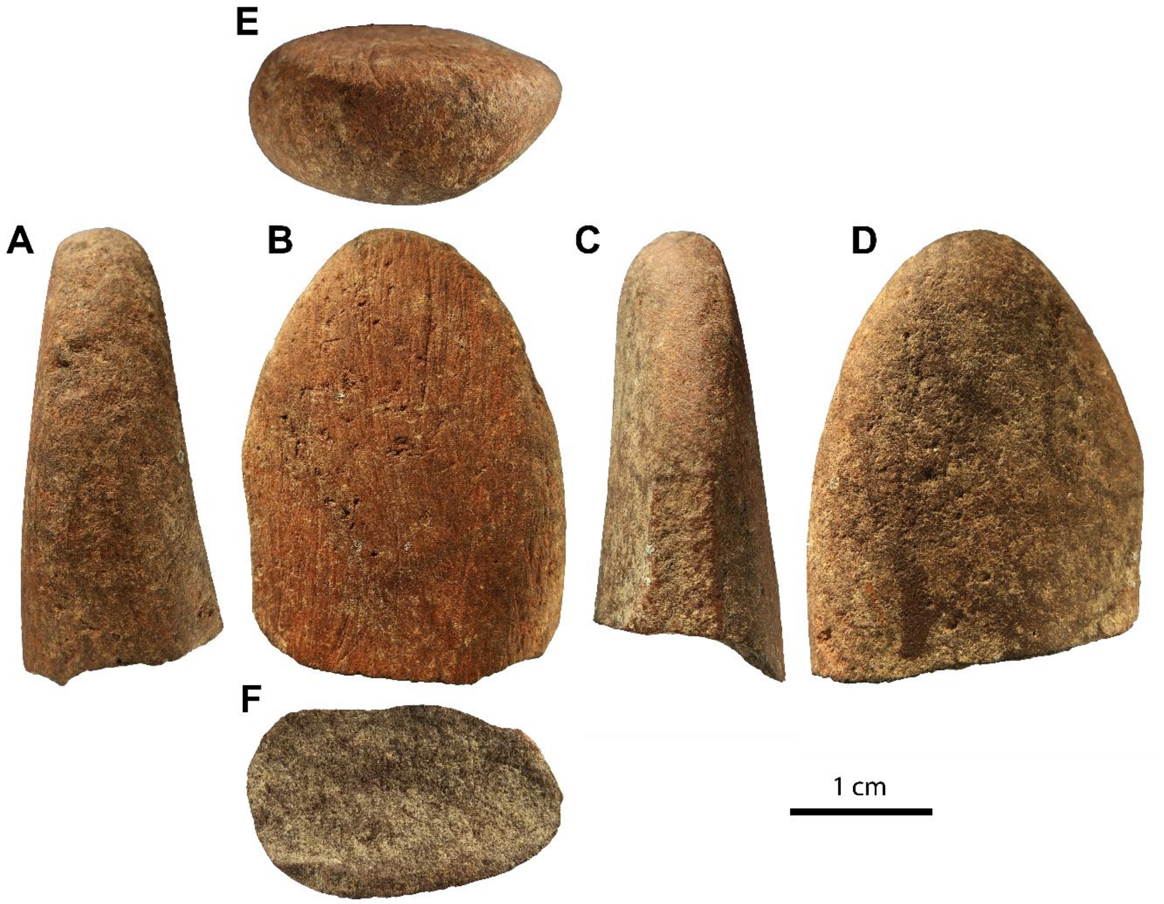

Figure S5: Artefact CB.200, showing six different aspects of the piece. Details B and D show the two flattened faces, one covered with longitudinal irregular striations covered with pecking marks (B) and the other showing impact marks from pecking (D). Detail a shows a lateral surface with irregular pecking marks, and detail C shows the other with oblique grinding striations on top of pecking marks. Detail E shows the working top with deep impact scars, detail F shows the bottom of the piece. Photo by F. d'Errico.

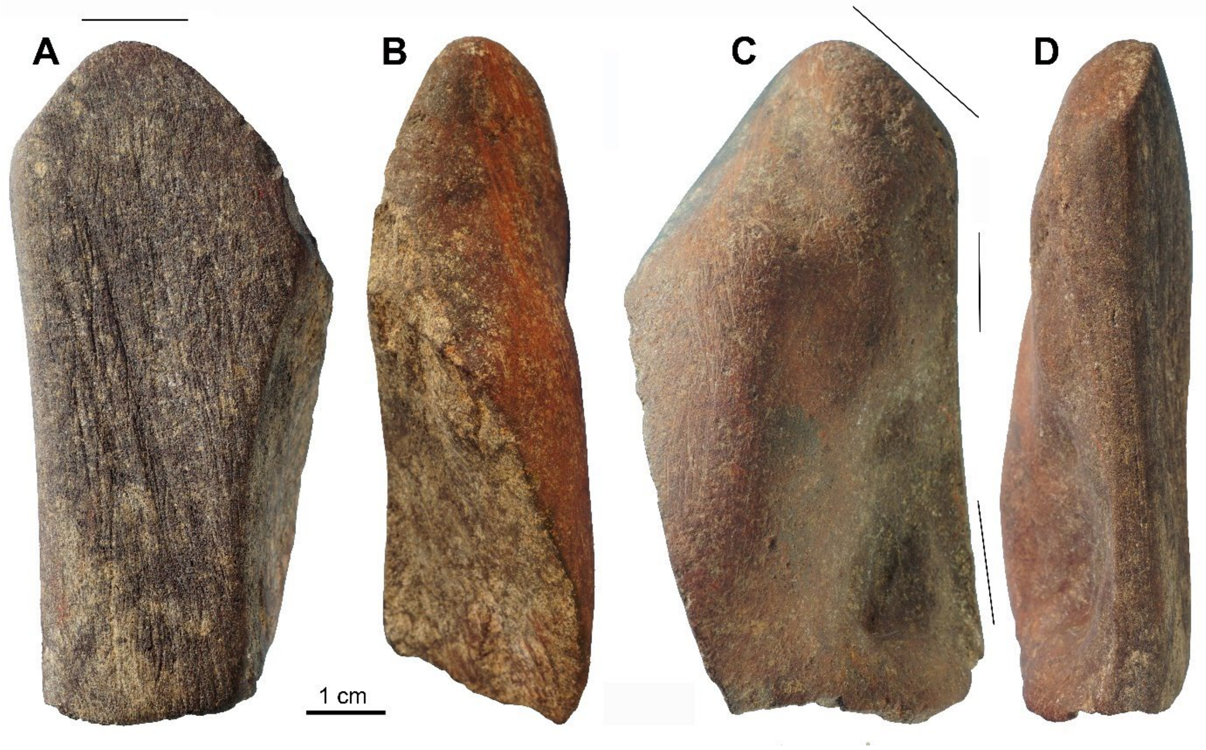

Figure S6: Artefact CFB-CFC.1566 showing four aspects. Detail A shows the faceted surface with irregular and grouped scraping striations, detail C shows the opposite site with irregular superficial striations, likely resulting from light scraping and general use. Detail B shows deep impact marks near the tip as well as two flake removals lower down, both of which may have been the result of pressure flaking. Detail D shows a longitudinal facet with striations, likely from grinding. Photo by F. d’Errico

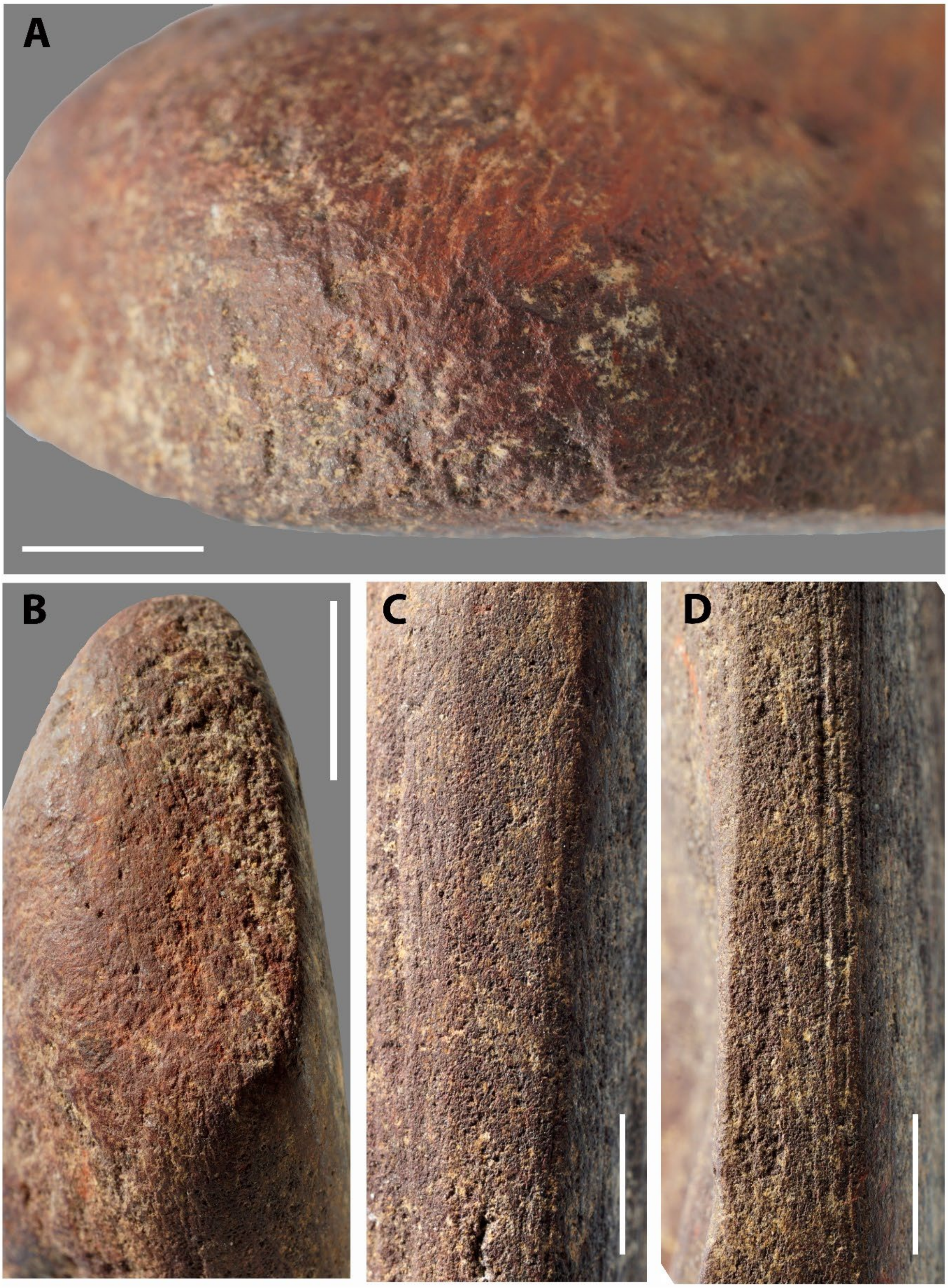

Figure S7: Artefact CFB-CFC.1566 macro images of use-traces. Detail A shows striations stemming from micro-chipping at the tip, detail B shows a ground facet with deep impact marks. Details C and D show a long fact running the length of the piece (fig. S6d) with superficial striations and overground peck marks higher up the piece (D) and deeper striations lower down the piece (detail D). All scales are 5 mm. Photo by F. d'Errico.

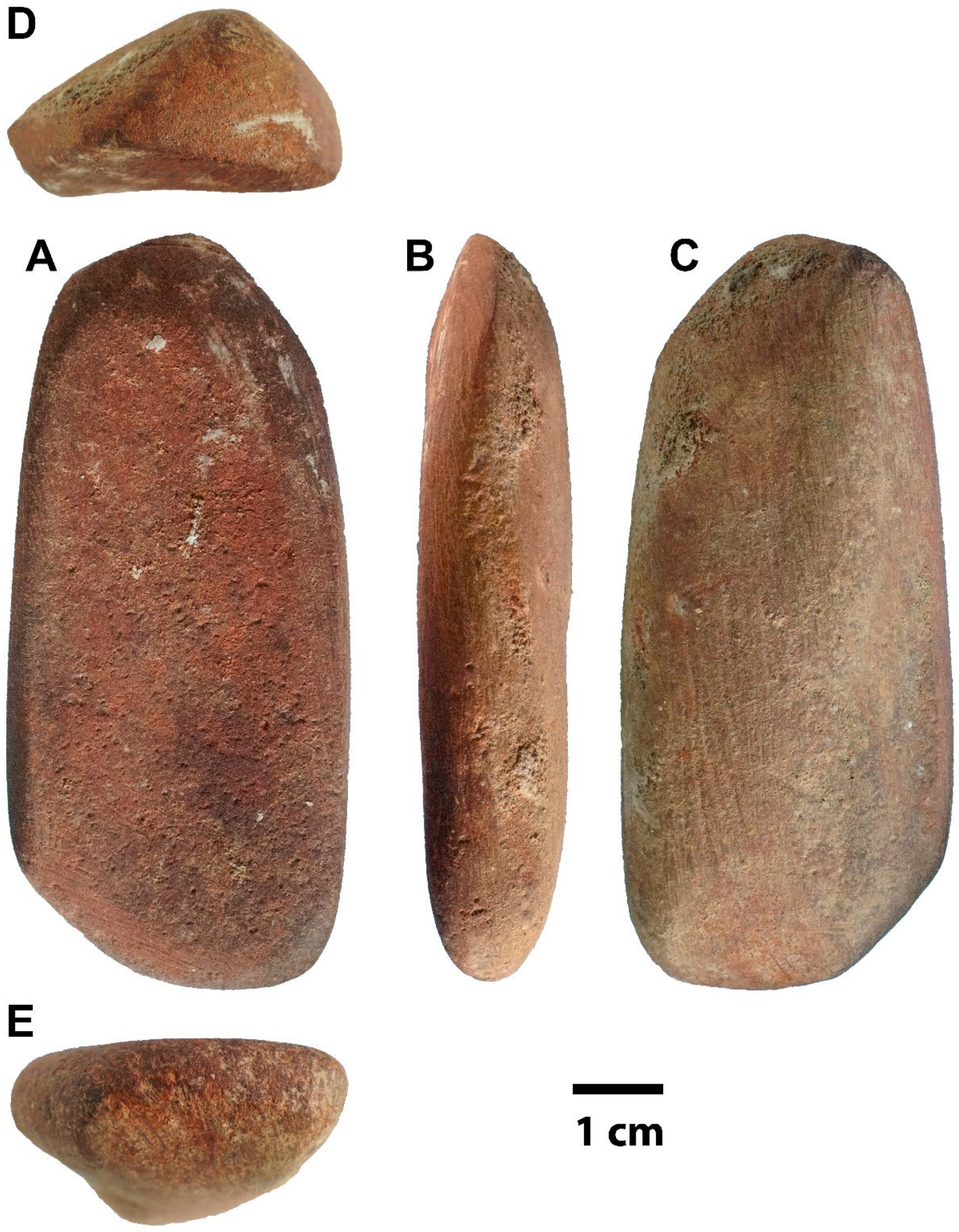

Figure S8: Artefact CFA-CFB-CFC.155 showing five aspects. Detail A shows a smoothed and slightly concave surface with irregularly dispersed peck marks, and is bordered by striated surfaces from light grinding. Detail B shows a lateral view with light striations from grinding, detail C shows a smoothed, shaped surface from light grinding. Detail D shows a striated facet resulting from tool use, and detail E shows deep impact scars also from tool use. Photo by F. d'Errico.

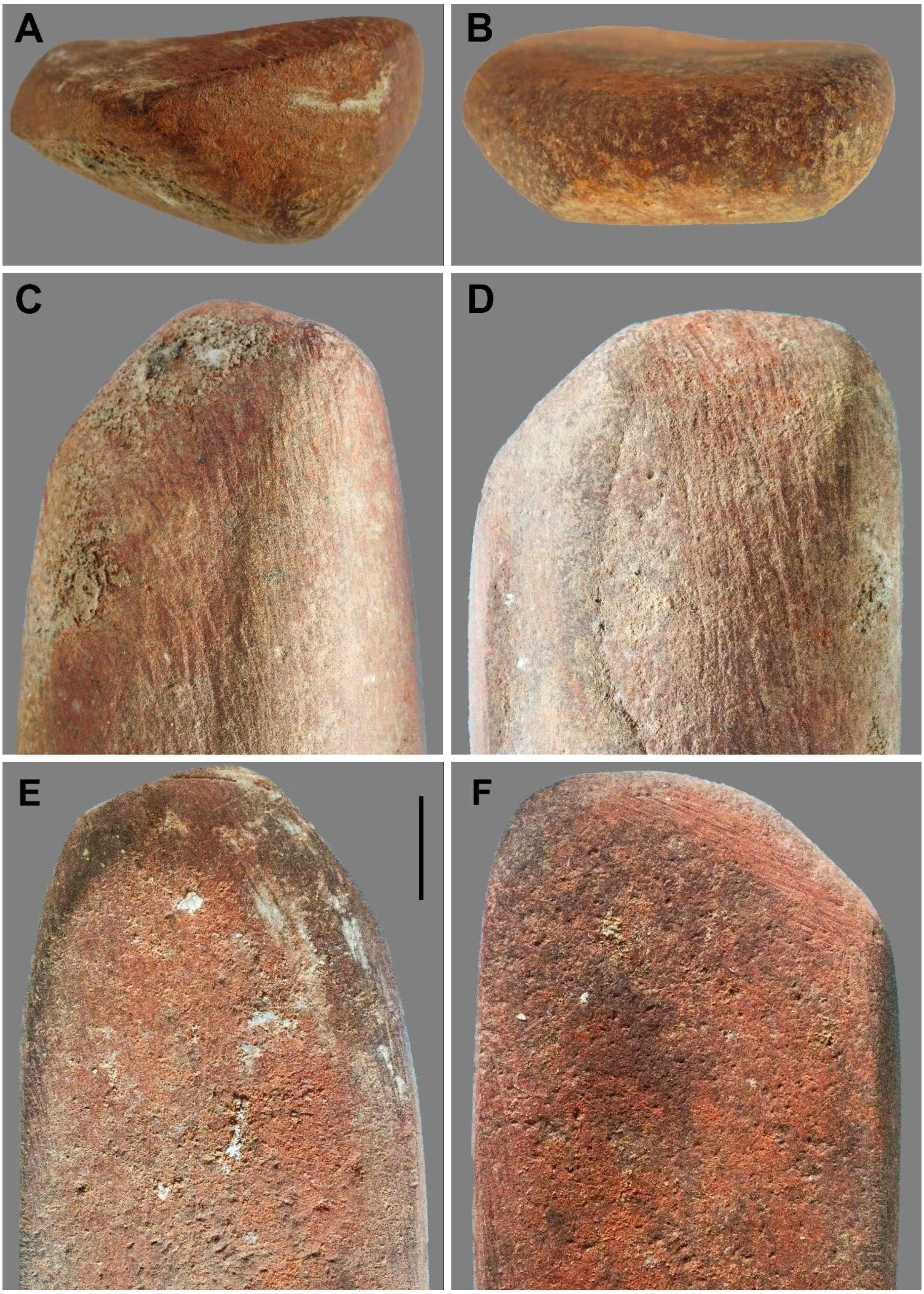

Figure S9: Macro images of use-wear on artefact CFA-CFB-CFC.155. Detail A shows a striated facet with some use-wear along edges, followed by deeper, elongated striations (detail C) suggesting use as a retoucher, detail B shows deeper impact scars oriented near one surface (right) suggesting its use by a right-handed person, as well as stemming striations from points of impact (detail D). Details E and F show top and bottom views of the concave surface (fig. S8a) exhibiting pecking marks and striated surfaces along the edges, likely from light grinding in order to regularize the shape. Scale is 1 cm. Photo by F. d'Errico.

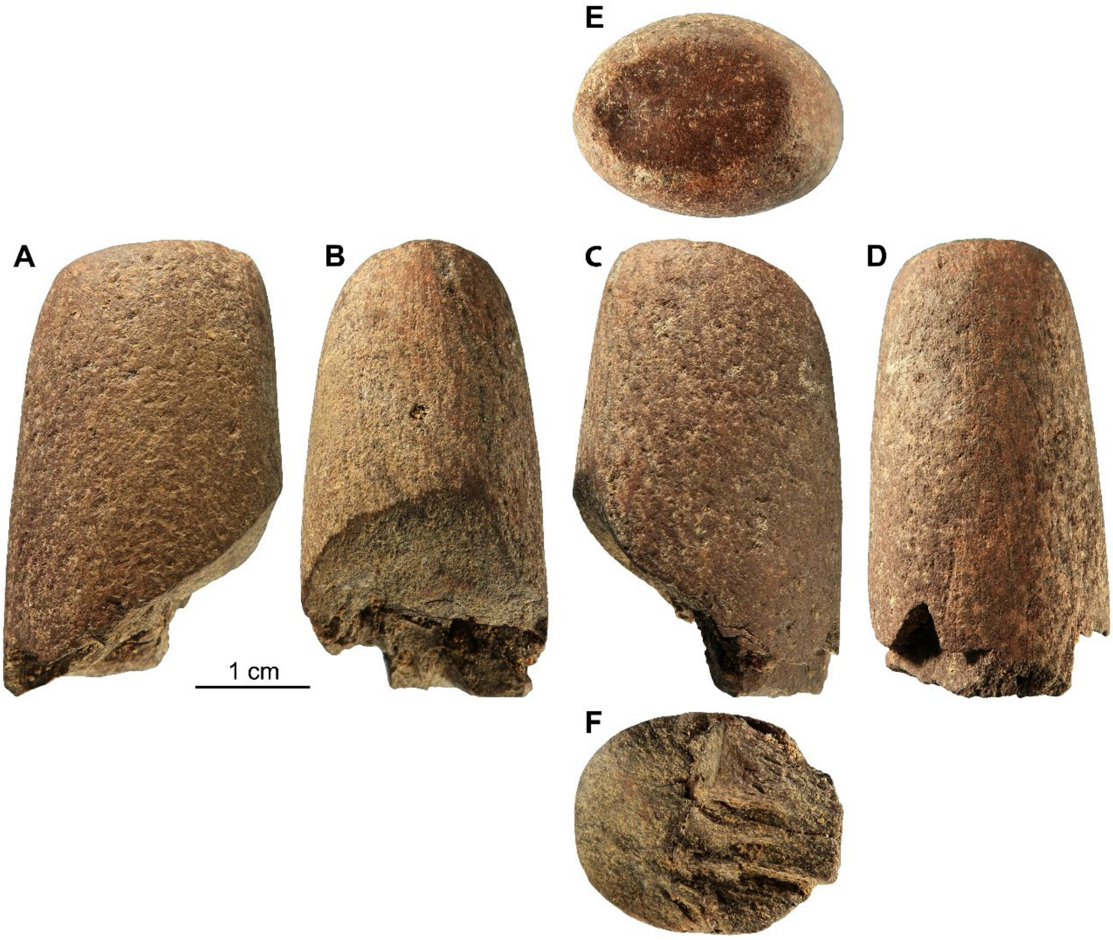

Figure S10: Artefact CH/CI.154 showing six aspects. Details A and C show numerous pecking marks dispersed over the broader surfaces of the piece, with details B and D showing striations consistent with grinding on the sides. Detail E shows the faceted, polished, elliptical surface at the tip with use-wear along with edges. Detail F shows the broken end of the piece. Photo by F. d'Errico

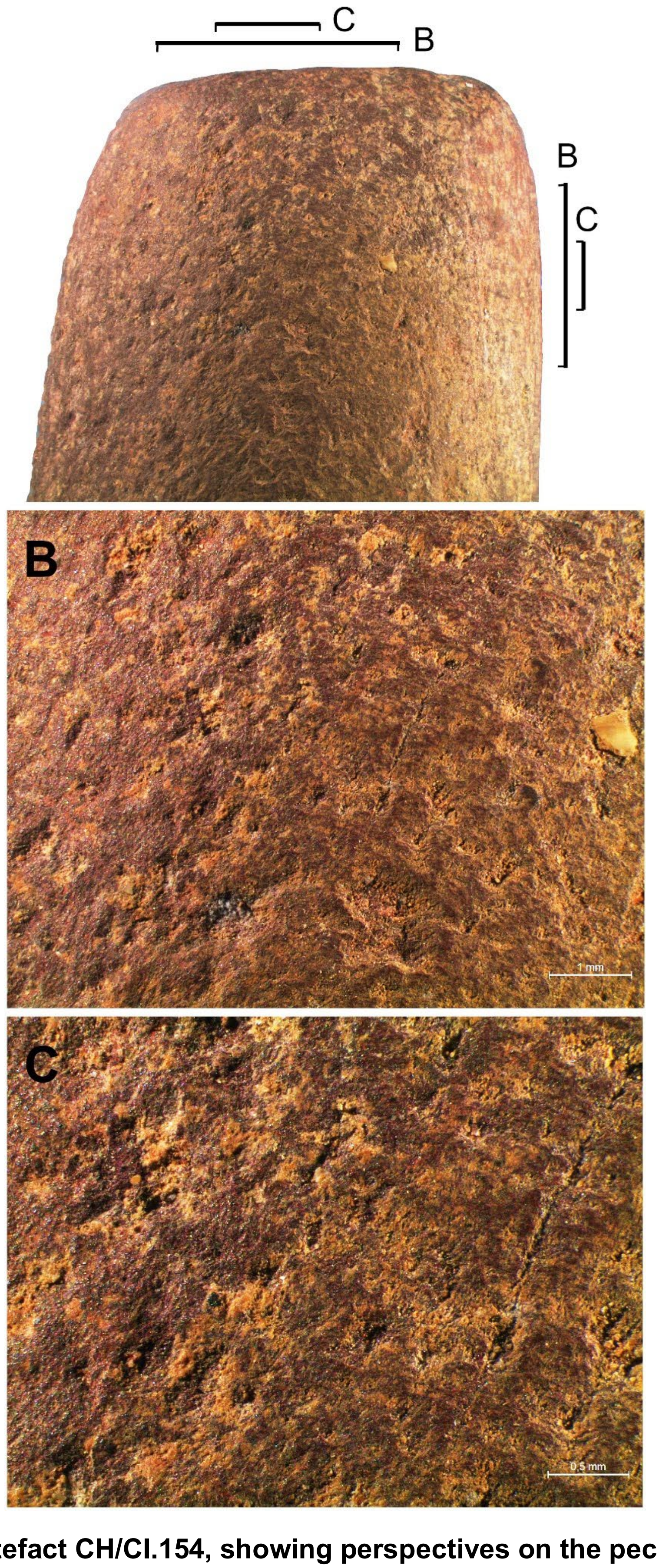

Figure S11: Artefact CH/CI.154, showing perspectives on the pecking marks used to shape the object. Photo by F. d’Errico.

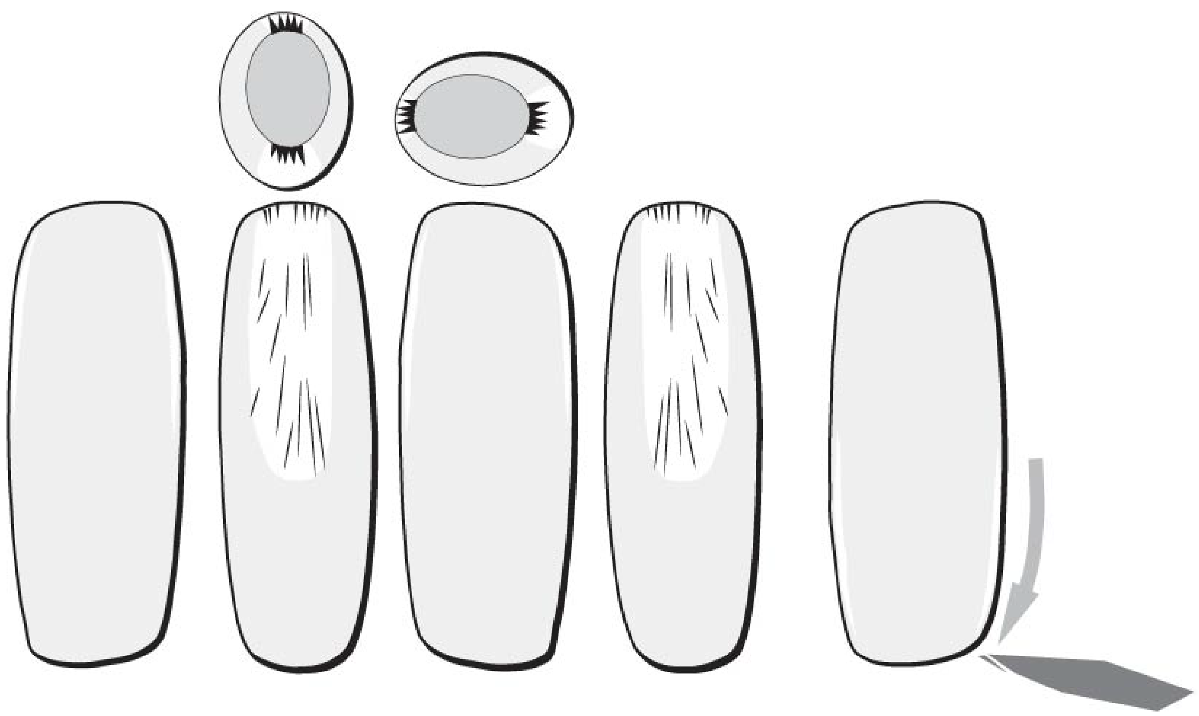

Figure S12: Schematic of Artefact CH/CI.154, showing possible original morphology. The light gray areas indicate the surface shaped by pecking, the dark gray area (top) shows the natural cortex at one end. In white are the facets covered by elongated striations produced by the repeated use of the pressure flaker; the pointed short striations identify the areas smoothed by use at the edge between the flat end that the facets. Drawing by F. d’Errico

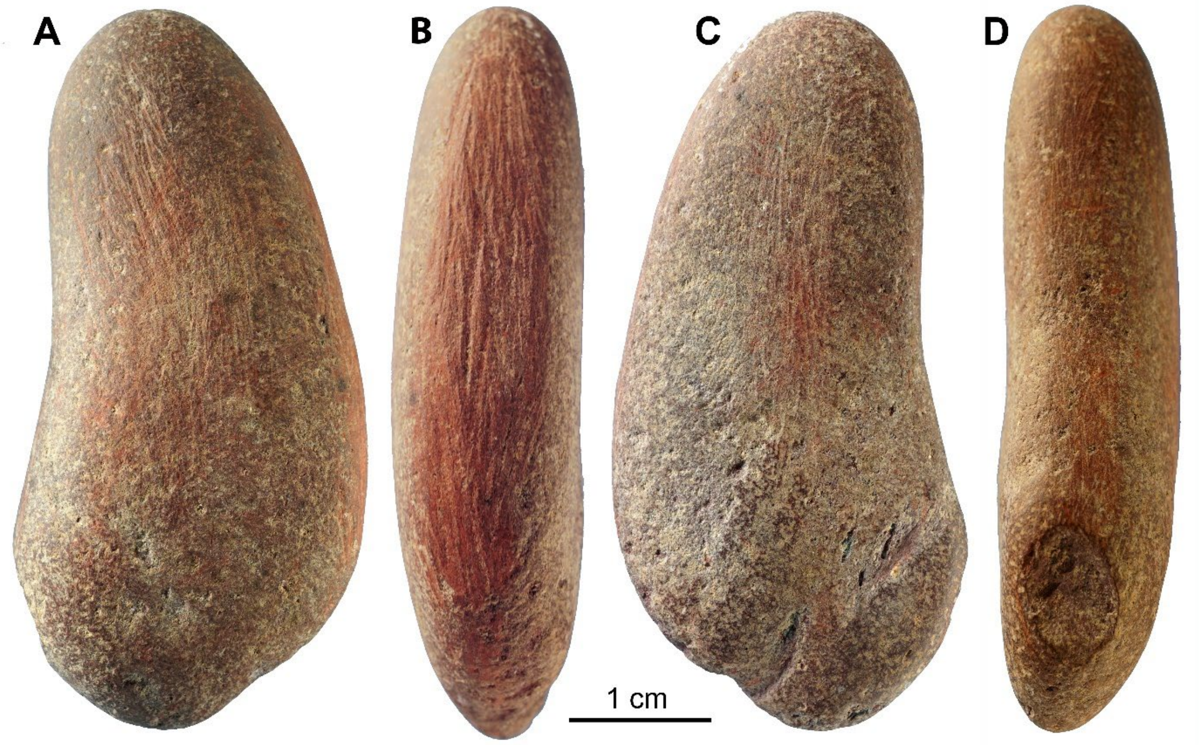

Figure S13: Artefact CH/CI.1562 showing four aspects. Details A and C show the broader surfaces with various impact marks, some with stemming striations, and some pecking near the tips. Detail B shows deep striations stemming from points of impact on the tip, as well as from scraping. Detail D shows some impact marks and light striations with a small removal near the bottom. Photo by F. d'Errico

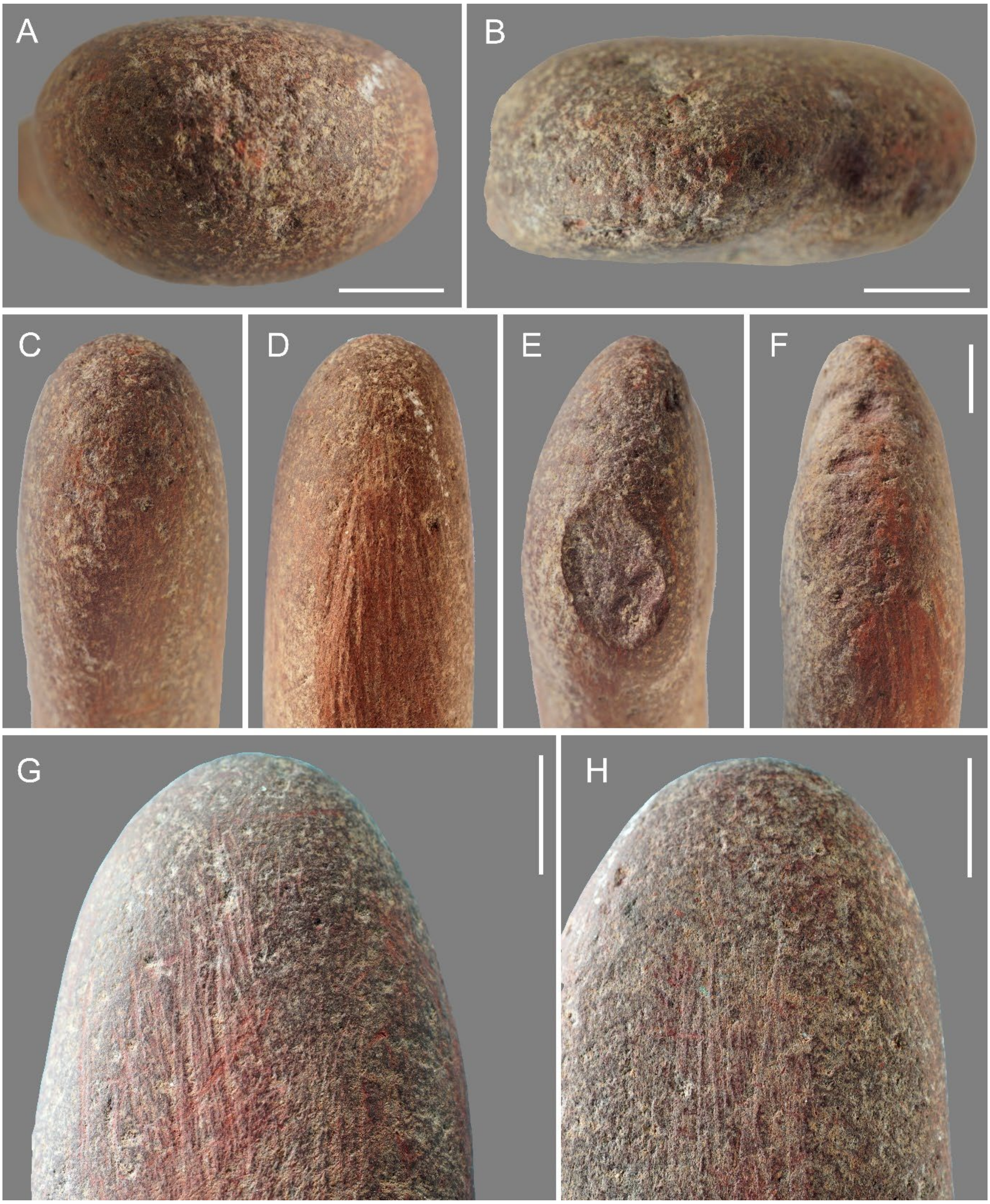

Figure S14: Macro images of use-wear on artefact CH/CI.1562. Details A and B show deep impact marks on either tip of the piece due to its use as a tool for retouching. Details C and D show either side following the tip from detail a, with striations stemming from point of impact in detail D. Details E and F show deep impact marks on the anterior end, with a removal that was likely the result of its use as a retoucher. Details G (tip of fig. S13a) and H (tip of fig. S13c) show light, irregular striations, likely from grinding, with some interspersed pecking marks and impact scars. All scales are 5 mm. Photo by F. d'Errico

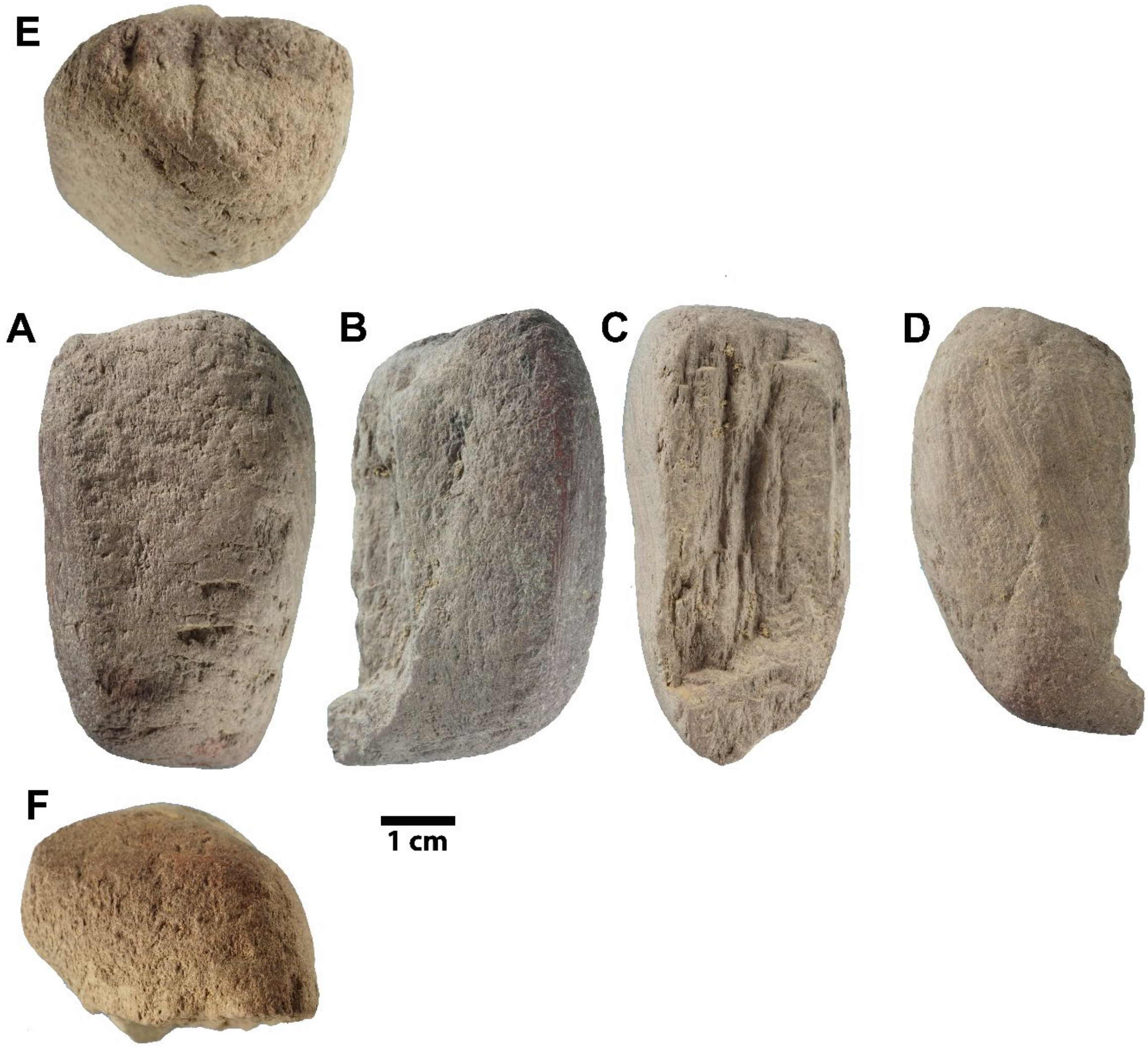

Figure S15: Artefact CH/CI2.480 showing six aspects. The broader surfaces are shown in details A and B, where pecking marks of various depths and sizes can be seen, as well as light, irregular scraping striations are oriented along the long axis. Detail C shows the natural breaking surface of the object with some light pecking marks along the end surfaces, detail D shows obliquely oriented scraping striations along two surfaces. Detail E shows one working end with deep, elongated impact marks, and detail F shows groups of deep pecking and impact marks. Photo by F. d’Errico.

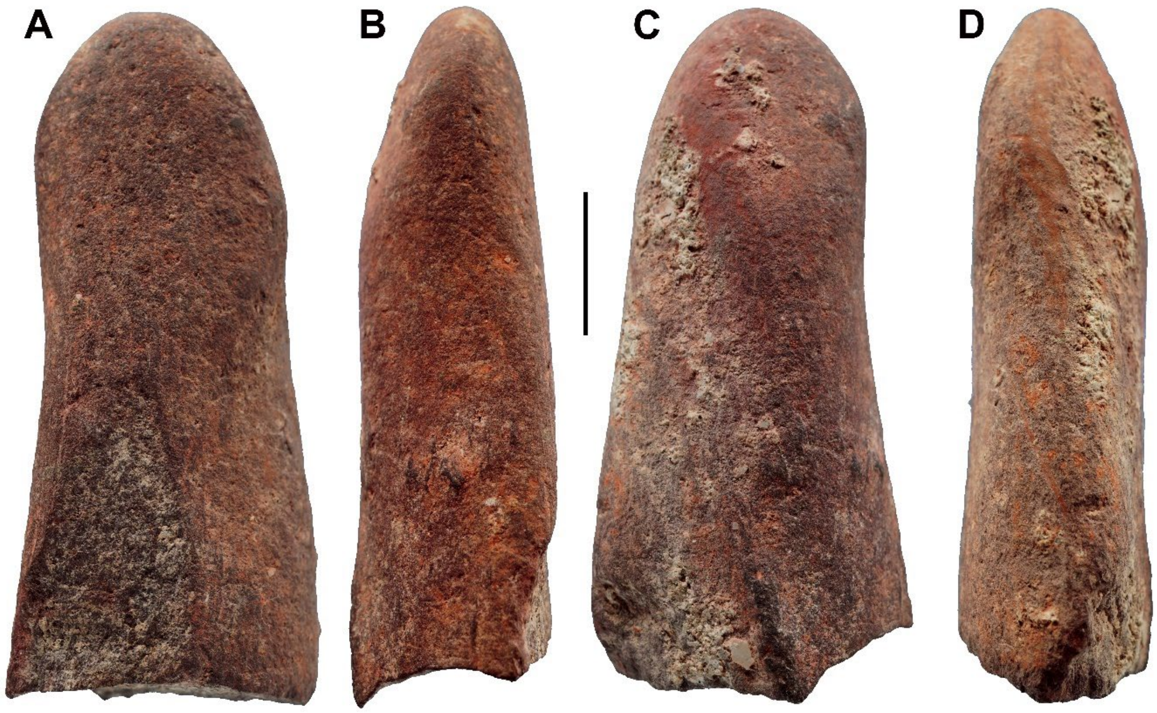

Figure S16: Artefact CP.485 showing four aspects. Details a and c show the broader surfaces with pecking marks (a) and superficial microstriations, offering evidence for rubbing (a and c), both of which likely helped to shape the object. Detail b shows irregular impact marks and detail d shows evidence for rubbing near the tip. Scale is 1 cm. Photo by F. d'Errico

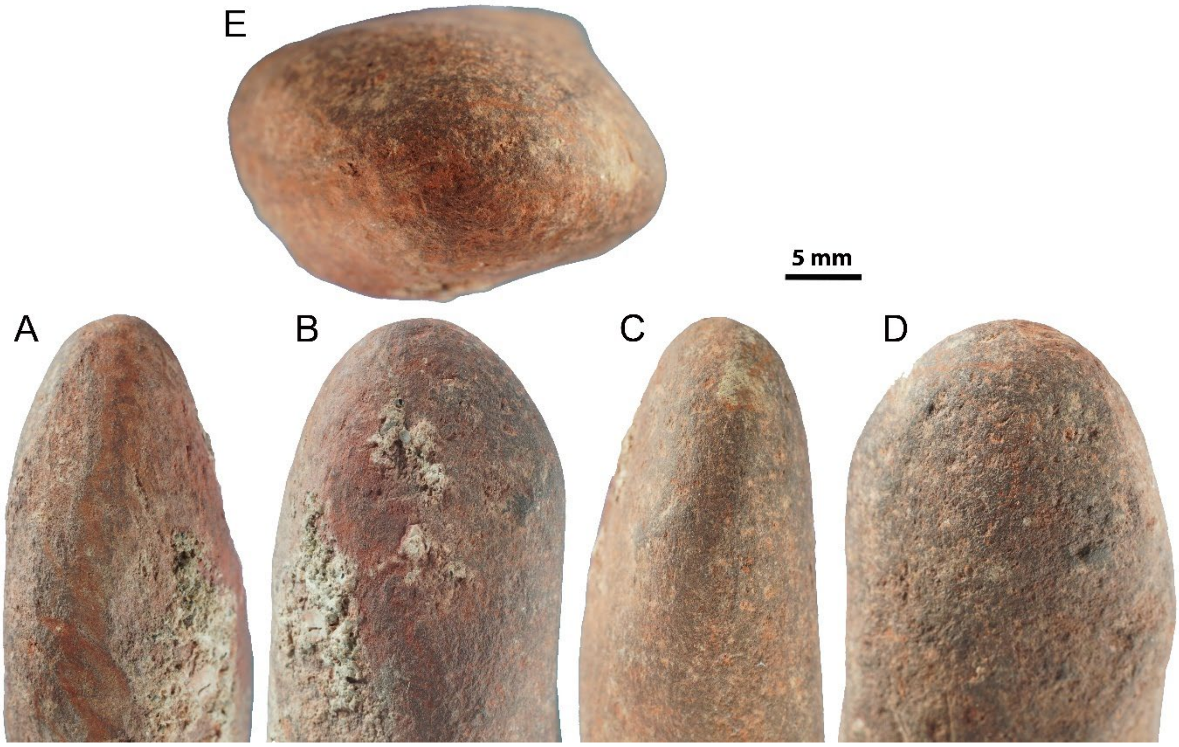

Figure S17: Macro images of use-wear on artefact CP.485. Detail A shows evidence for rubbing, possibly to rejuvenate the piece, and detail B shows deep, irregular impact scars and evidence for rubbing. Details C and D show irregular pecking marks with some light stemming striations, and detail E shows some light impact and pecking marks along with superficial striations. Photo by F. d'Errico

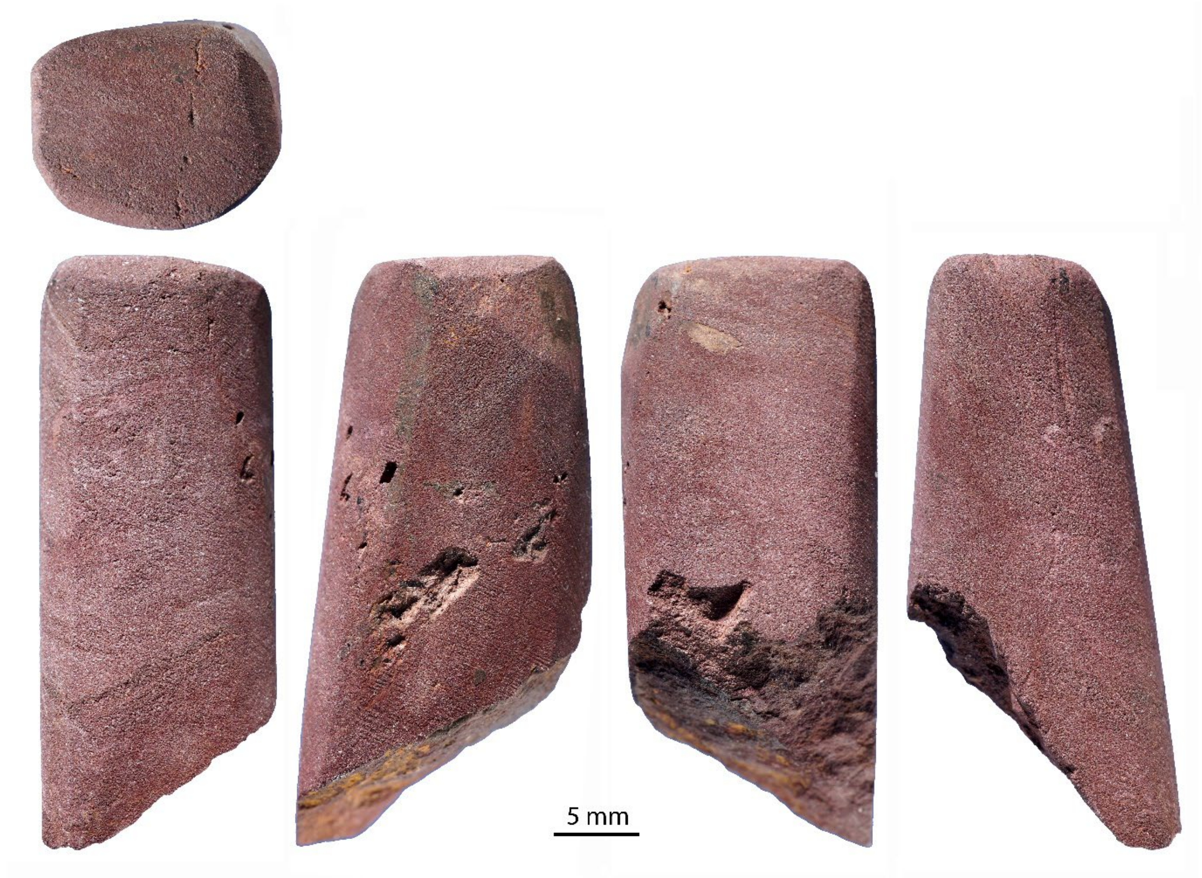

Figure S18: Image showing experimentally replicated piece EXR01 before use. Photo by F. d'Errico

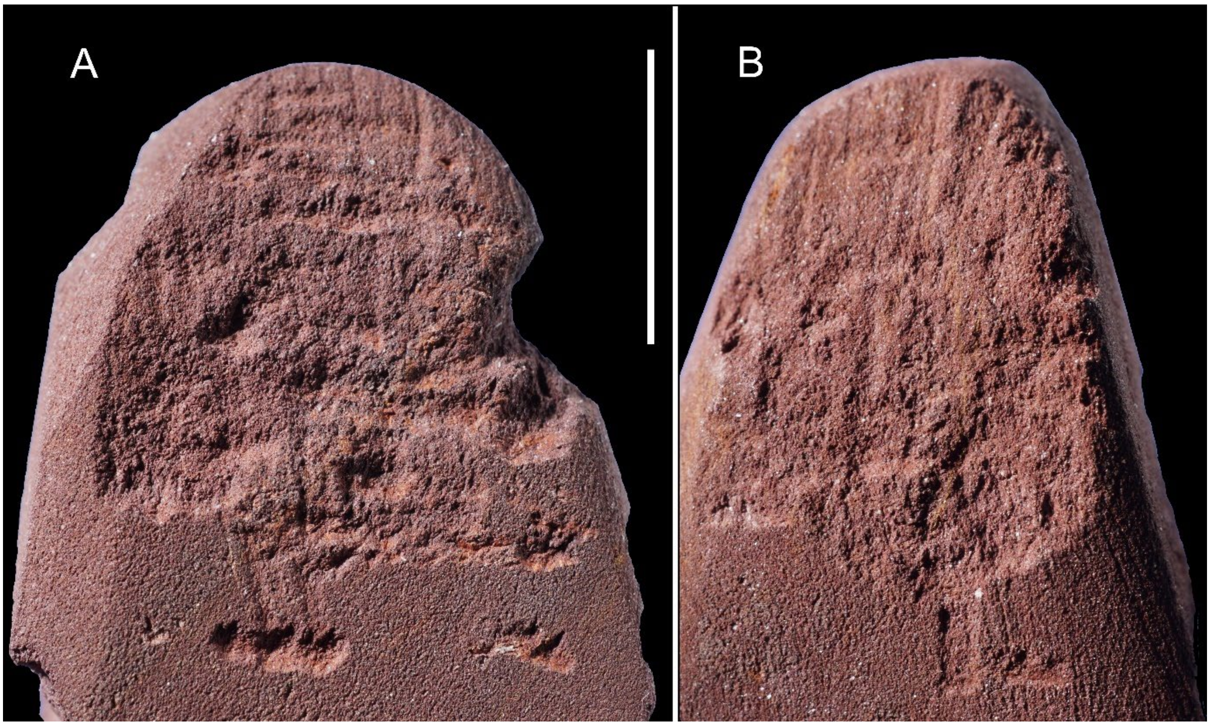

Figure S19: EX02, with traces produced by horizontal retouching. Photo by F. d'Errico

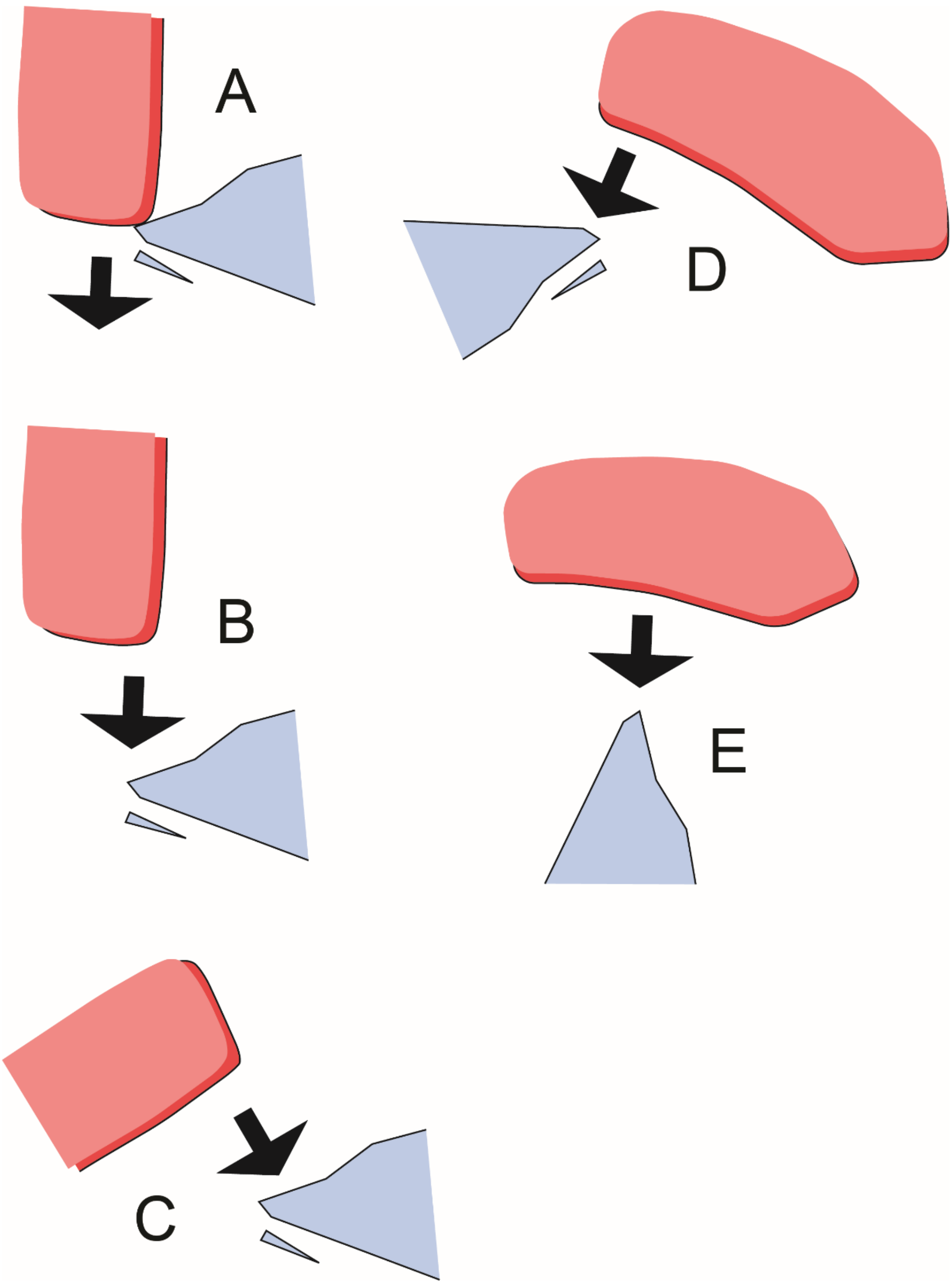

Figure S20: Drawing of experimental actions used. Techniques shown in A-C were replicated three times on the EX01. The details are as follows: A) EX01 being used as a pressure flaker, B) to retouch a flake edge by percussion following a direction parallel to the object main axis, i.e. by keeping the object vertical, C) to retouch a flake edge by percussion with the main axis of the object kept perpendicular or slightly angled to the direction of the movement (tangential) with an elongated ochre piece (EXR01); D) detail showing EX02 being used as a retoucher, and E) percussing the surface of EX02 on the pointed end of the silcrete flake, to peck the flat surface of the ochre with the flake tip. Drawing by F. d'Errico.

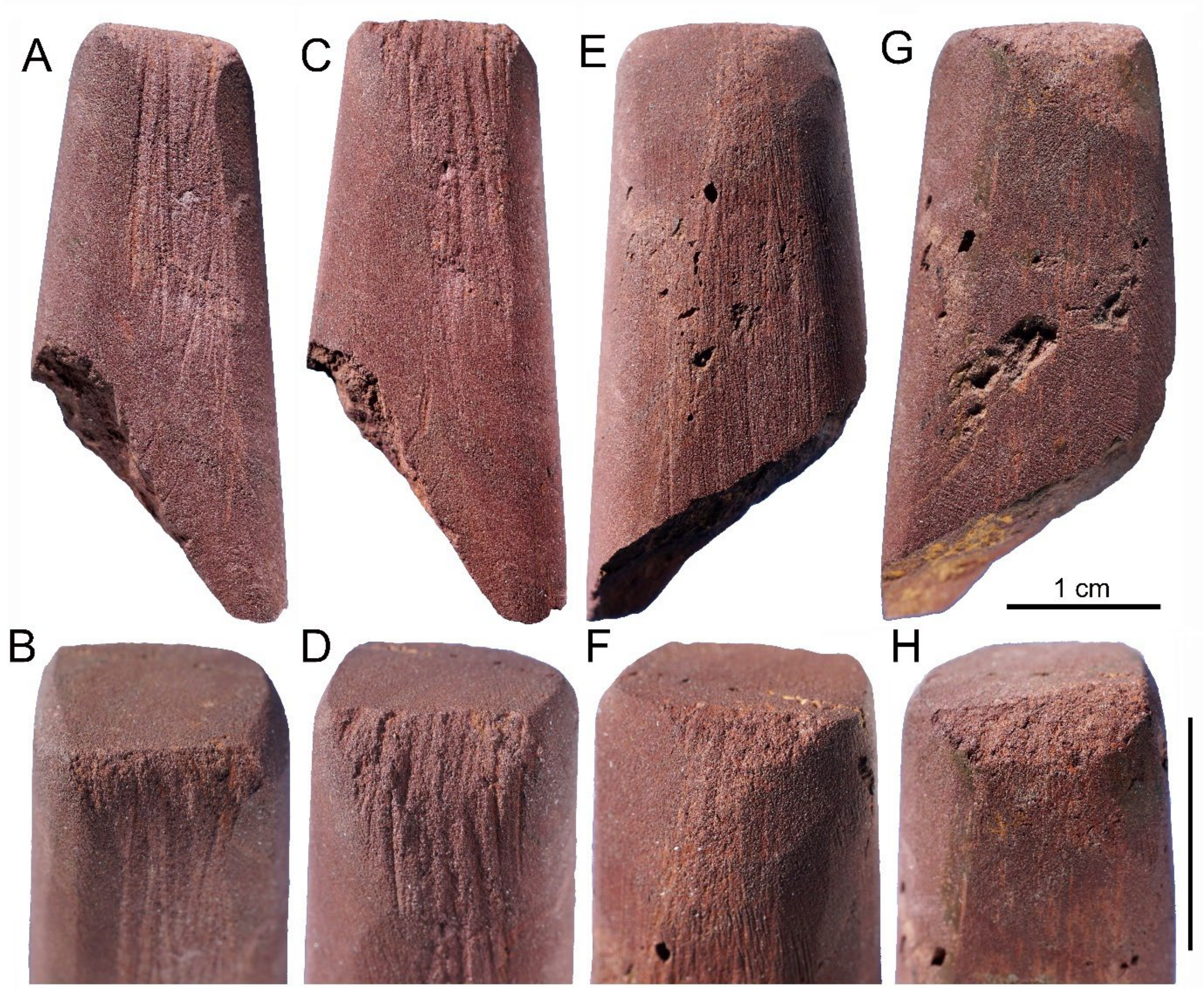

Figure S21: Images of EX01 post-experiment. Details A-D show two examples of use-traces resulting from pressure flaking. Details E-H show results of the use as a retoucher by percussion, keeping the ochre tool vertical. Photo by F. d’Errico

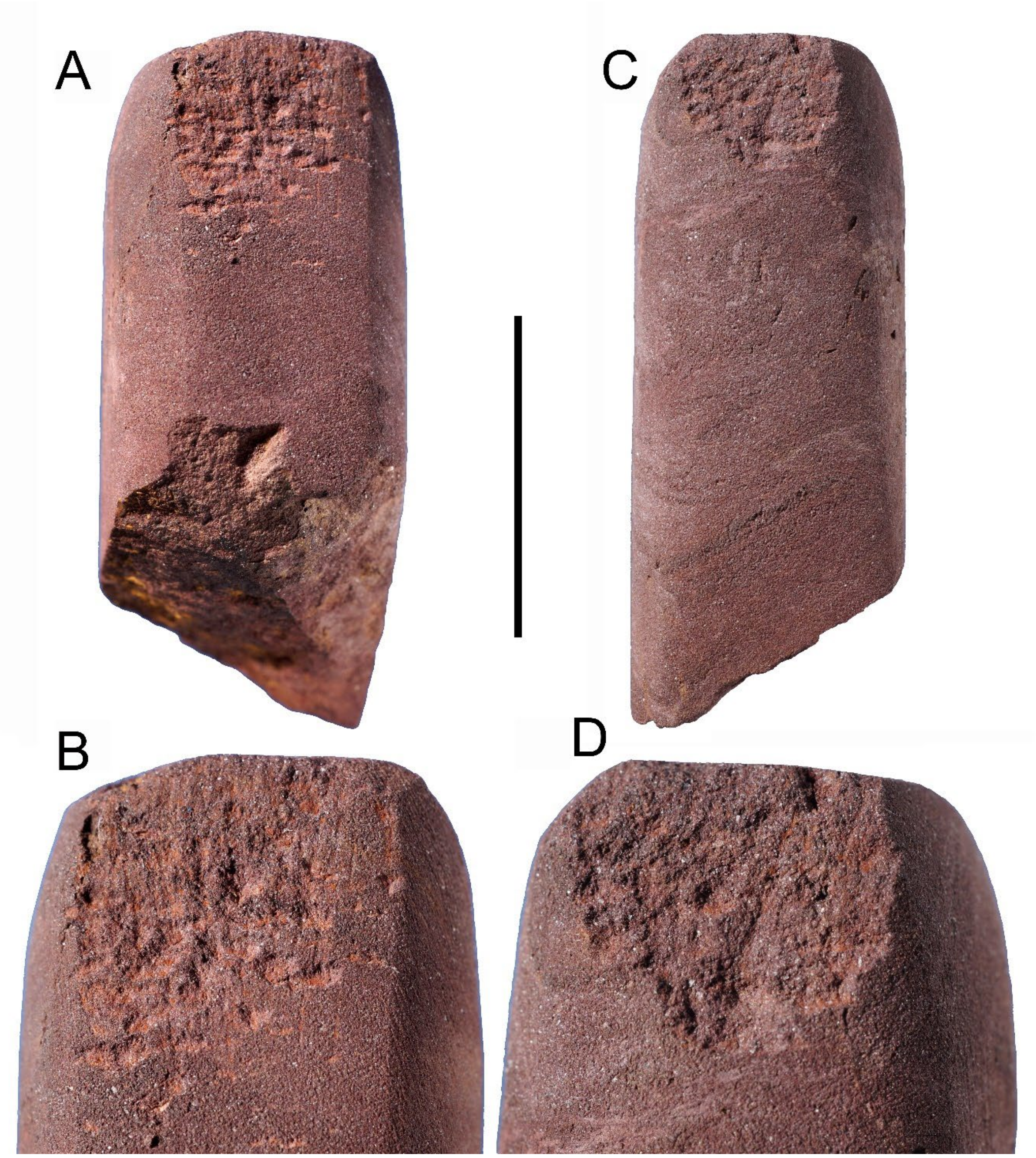

Figure S22: EX01 post-experiment. The images show examples of use-traces resulting from its use as a retoucher that was kept horizontal during the retouching process. Detail A shows deep, horizontally oriented impact marks with stemming striations, detail B is a close-up of this side. Detail C shows deep, irregular impact marks from retouching and D is a close-up of these marks. Photo by F. d'Errico

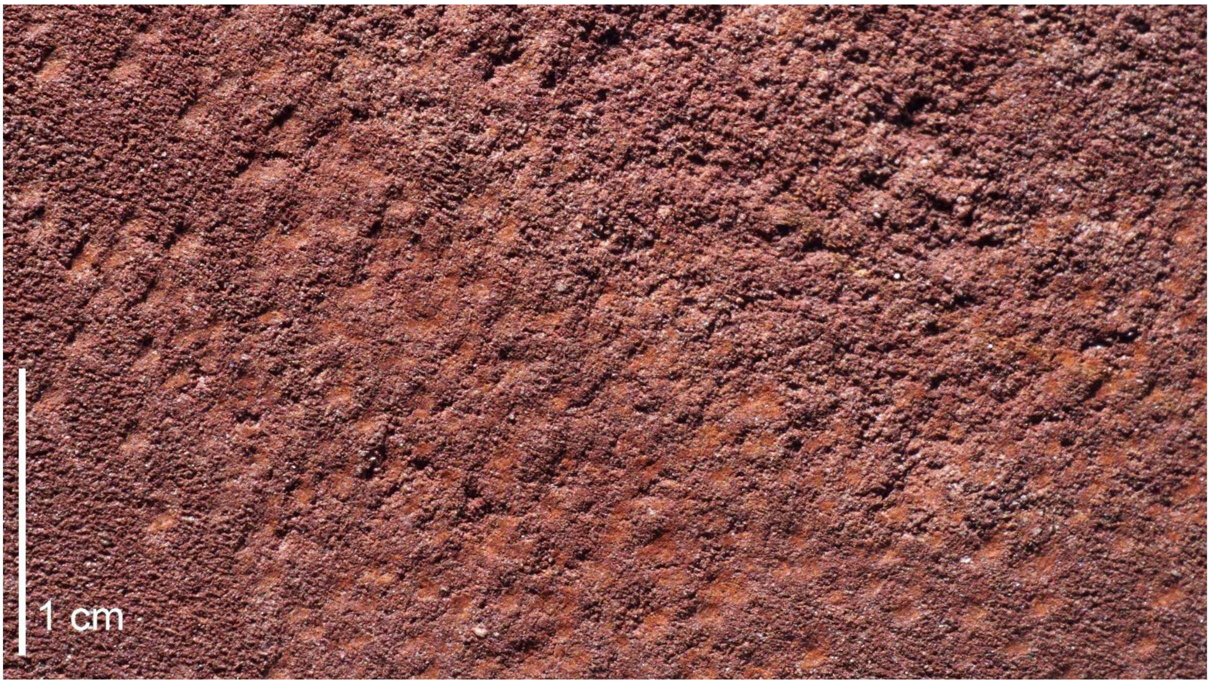

Figure S23: Experimental pecking traces shown on EX02. Photo by F. d’Errico

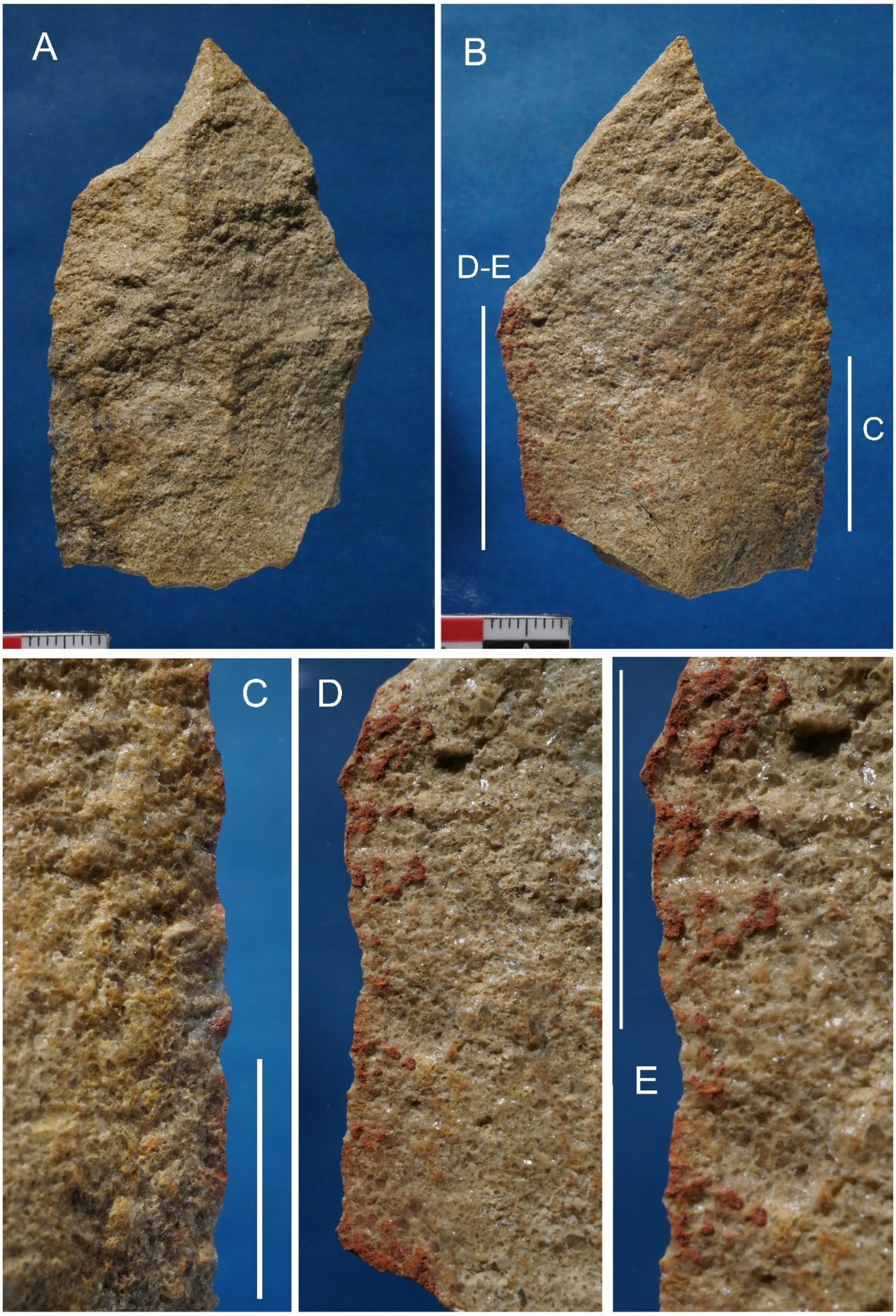

SI Figure S24: Silcrete flake experimentally retouched. The details show the retouch by pressure-flaking on one edge (C) and the other edge (D-E) by percussion. Notice the more abundant ochre residues left on the edge by the latter technique. Scales in C and D = 1 cm. Photos by F. d'Errico.

Table S1: Stratigraphic and temporal contexts for the seven identified ochre retouchers from BBC.

UNIT	ID	QUAD	PHASE	UNIT AGE	Size (mm)	Weight (g)	Figure	Type
CB	200	F7b	M1	67 ± 7 (CA; TL min. age)<br>81 ± 10 (CA; TL max. age)<br><br>68 ± 6 (CC; TL min. age)<br>82 ± 8 (CC; TL max. age)	40.93 x 32.13 x 16.15	36.12	Fig. 3A-G;<br>fig. S5	Indurated shale
CFB-CFC	1566	G4d	M2 UP	77.5 ± 3.8 (OSL)<br>69.0 ± 3.4 (OSL)	75.83 x 40.92 x 19.9	110.3	figs. S6, S7	Indurated shale
CFA-CFB-CFC	155	H7c	M2 UP	74.0 ± 3.9 (OSL)<br>77.5 ± 3.8 (OSL)<br>69.0 ± 4.0 (OSL)	78.56 x 35.6 x 15.66	79.53	figs. S8, S9	Indurated shale
CH/CI	154	G7a	M3	88.2 ± 5.3 (OSL)	34.5 x 21.4 x 19	24.38	Fig. 3H-J;<br>figs. S10-S12	Indurated shale
CH/CI	1562	H4c	M3	88.2 ± 5.3 (OSL)	47.16 x 23.44 x 11.74	21.1	figs. S13-S14	Indurated shale
CH/CI2	480	I6b	M3	88.2 ± 5.3 (OSL)	58.06 x 29.68 x 32.38	76.8	fig. S15	Indurated shale
CP	485	H6a	M3	90.9 ± 4.3 (OSL)	45.44 x 21.43 x 12.81	17	figs. S16-S17	Coarse hematic shale

REFERENCES AND NOTES

1. I. Watts, M. Chazan, J. Wilkins, Early evidence for brilliant ritualized display: Specularite use in the Northern Cape (South Africa) between □500 and □300 Ka. *Curr. Anthropol.* **57**, 287–310 (2016).

2. A. Brooks, J. Yellen, A. Zipkin, L. Dussubieux, P. Rick, in *The 81st Annual Meeting of the Society for American Archaeology* (Society for American Archaeology, 2016), vol. 53.

3. R. Dapschauskas, M. B. Göden, C. Sommer, A. W. Kandel, The emergence of habitual ochre use in Africa and its significance for the development of ritual behavior during the Middle Stone Age. *J. World Prehist.* **35**, 233–319 (2022).

4. D. E. Rosso, The first uses of colour: What do we know? *J. Anthropol. Sci.* **100**, 45–69 (2022).

5. F. d'Errico, H. Salomon, C. Vignaud, C. Stringer, Pigments from the Middle Palaeolithic levels of Es-Skhul (Mount Carmel, Israel). *J. Archaeol. Sci.* **37**, 3099–3110 (2010).

6. W. Roebroeks, M. J. Sier, T. K. Nielsen, D. De Loecker, J. M. Parés, C. E. S. Arps, H. J. Mücher, Use of red ochre by early Neandertals. *Proc. Natl. Acad. Sci. U.S.A.* **109**, 1889–1894 (2012).

7. L. Dayet, J.-P. Faivre, F.-X. Le Bourdonnec, E. Discamps, A. Royer, É. Claud, C. Lahaye, N. Cantin, E. Tartar, A. Queffelec, B. Gravina, A. Turq, F. d'Errico, Manganese and iron oxide use at Combe-Grenal (Dordogne, France): A proxy for cultural change in Neanderthal communities. *J. Archaeol. Sci. Rep.* **25**, 239–256 (2019).

8. H. De Lumley, F. Audubert, S. Khatib, C. Perrenoud, B. Roussel, T. Saos, A. Szelewa, "Les crayons d'ocre du site acheuléen de Terra Amata," in *Terra Amata Nice Alpes-Maritimes, France Tome V*, H. De Lumley, Ed. (CNRS Editions, 2016), chap. 44, pp. 233–277. [The ochre 'crayons' of the Acheulean site of Terra Amata].

9. F. d'Errico, C. S. Henshilwood, "The origin of symbolically mediated behaviour," in *Homo symbolicus: The Dawn of Language, Imagination and Spirituality*, C. S. Henshilwood, F. d'Errico, Eds. (John Benjamins Publishing Company, 2011), pp. 49–74.

10. S. McBrearty, A. S. Brooks, The revolution that wasn't: A new interpretation of the origin of modern human behavior. *J. Hum. Evol.* **39**, 453–563 (2000).

11. R. F. Rifkin, L. Dayet, A. Queffelec, B. Summer, M. Lategan, F. d'Errico, Evaluating the photoprotective effects of ochre on human skin by in vivo SPF assessment: Implications for human evolution, adaptation and disperal. *PLOS ONE* **10**, e0136090 (2015).

12. R. F. Rifkin, Assessing the efficacy of red ochre as a prehistoric hide tanning ingredient. *J. Afr. Archaeol.* **9**, 131–158 (2011).

13. L. Wadley, Compound-adhesive manufacture as a behavioral proxy for complex cognition in the Middle Stone Age. *Curr. Anthropol.* **51**, S111–S119 (2010).

14. L. Wadley, T. Hodgskiss, M. Grant, Implications for complex cognition from the hafting of tools with compound adhesives in the Middle Stone Age, South Africa. *Proc. Natl. Acad. Sci. U.S.A.* **106**, 9590–9594 (2009).

15. I. Watts, "The origin of symbolic culture: The Middle Stone Age of southern Africa and Khoisan ethnography," thesis, University of London, London (1998).

16. W. H. I. Bleek, L. Lloyd, *Specimens of Bushmen Folklore* (G. Allen & Company Ltd., 1911).

17. F. d'Errico, A. Pitarch Martí, C. Shipton, E. Le Vraux, E. Ndiema, S. Goldstein, M. D. Petraglia, N. Boivin, Trajectories of cultural innovation from the Middle to Later Stone Age in eastern Africa: Personal ornaments, bone artifacts, and ocher from Panga ya Saidi, Kenya. *J. Hum. Evol.* **141**, 102737 (2020).

18. A. S. Brooks, J. E. Yellen, R. Potts, A. K. Behrensmeyer, A. L. Deino, D. E. Leslie, S. H. Ambrose, J. R. Ferguson, F. d'Errico, A. M. Zipkin, S. Whittaker, J. Post, E. G. Veatch, K. Foecke, J. B. Clark, Long-distance stone transport and pigment use in the earliest Middle Stone Age. *Science* **360**, 90–94 (2018).

19. G. H. Odell, The morphological express at function junction: Searching for meaning in lithic tool types. *J. Anthropol. Res.* **37**, 319–342 (1981).

20. C. S. Henshilwood, F. d'Errico, K. L. van Niekerk, Y. Coquinot, Z. Jacobs, S.-E. Lauritzen, M. Menu, R. García-Moreno, A 100,000-year-old ochre-processing workshop at Blombos Cave, South Africa. *Science* **334**, 219–222 (2011).

21. Z. Jacobs, B. G. Jones, H. C. Cawthra, C. S. Henshilwood, R. G. Roberts, The chronological, sedimentary and environmental context for the archaeological deposits at Blombos Cave, South Africa. *Quat. Sci. Rev.* **235**, 105850 (2020).

22. M. Vanhaeren, F. d'Errico, K. L. van Niekerk, C. S. Henshilwood, R. M. Erasmus, Thinking strings: Additional evidence for personal ornament use in the Middle Stone Age at Blombos Cave, South Africa. *J. Hum. Evol.* **64**, 500–517 (2013).

23. C. S. Henshilwood, F. d'Errico, R. Yates, Z. Jacobs, C. Tribolo, G. A. T. Duller, N. Mercier, J. C. Sealy, H. Valladas, I. Watts, A. G. Wintle, Emergence of modern human behavior: Middle Stone Age engravings from South Africa. *Science* **295**, 1278–1280 (2002).

24. C. S. Henshilwood, F. d'Errico, K. L. van Niekerk, L. Dayet, A. Queffelec, L. Pollarolo, An abstract drawing from the 73,000-year-old levels at Blombos Cave, South Africa. *Nature* **562**, 115–118 (2018).

25. C. S. Henshilwood, F. d'Errico, I. Watts, Engraved ochres from the Middle Stone Age levels at Blombos Cave, South Africa. *J. Hum. Evol.* **57**, 27–47 (2009).

26. P. Villa, M. Soressi, C. S. Henshilwood, V. Mourre, The Still Bay points of Blombos Cave (South Africa). *J. Archaeol. Sci.* **36**, 441–460 (2009).

27. V. Mourre, P. Villa, C. S. Henshilwood, Early use of pressure flaking on lithic artifacts at Blombos Cave, South Africa. *Science* **330**, 659–662 (2010).

28. K. S. Brown, C. W. Marean, A. I. R. Herries, Z. Jacobs, C. Tribolo, D. Braun, D. L. Roberts, M. C. Meyer, J. Bernatchez, Fire as an engineering tool of early modern humans. *Science* **325**, 859–862 (2009).

29. S. Soriano, P. Villa, L. Wadley, Ochre for the toolmaker: Shaping the Still Bay points at Sibudu (KwaZulu-Natal, South Africa). *J. Afr. Archaeol.* **7**, 41–54 (2009).

30. V. Rots, C. Lentfer, V. C. Schmid, G. Porraz, N. J. Conard, Pressure flaking to serrate bifacial points for the hunt during the MIS5 at Sibudu Cave (South Africa). *PLOS ONE* **12**, e0175151 (2017).

31. A. Högberg, M. Lombard, Still Bay point-production strategies at Hollow Rock Shelter and Umhlatuzana Rock Shelter and knowledge-transfer systems in southern Africa at about 80-70 thousand years ago. *PLOS ONE* **11**, e0168012 (2016).

32. P. Schmidt, A. Högberg, Heat treatment in the Still Bay-A case study on Hollow Rock Shelter, South Africa. *J. Archaeol. Sci. Rep.* **21**, 712–720 (2018).

33. E. Turner, L. Humphrey, A. Bouzouggar, N. Barton, Bone retouchers and technological continuity in the Middle Stone Age of North Africa. *PLOS ONE* **15**, e0230642 (2020).

34. F. d'Errico, L. R. Backwell, L. Wadley, Identifying regional variability in Middle Stone Age bone technology: The case of Sibudu Cave. *J. Archaeol. Sci.* **39**, 2479–2495 (2012).

35. I. Watts, "Red ochre, body painting, and language: Interpreting the Blombos ochre," in *The Cradle of Language*, R. Botha, C. Knight, Eds. (Oxford Univ. Press, 2009), pp. 93–129.

36. A. Hoegberg, M. Lombard, Indications of pressure flaking more than 70 thousand years ago at Umhlatuzana Rock Shelter. *S. Afr. Archaeol. Bull.* **71**, 53–59 (2016).

37. M. Vanhaeren, L. Wadley, F. d'Errico, Variability in Middle Stone Age symbolic traditions: The marine shell beads from Sibudu Cave, South Africa. *J. Archaeol. Sci. Rep.* **27**, 101893 (2019).

38. F. d'Errico, M. Vanhaeren, K. Van Niekerk, C. S. Henshilwood, R. M. Erasmus, Assessing the accidental versus deliberate colour modification of shell beads: A case study on perforated *Nassarius kraussianus* from Blombos Cave Middle Stone Age levels. *Archaeometry* **57**, 51–76 (2015).

39. F. d'Errico, M. Vanhaeren, L. Wadley, Possible shell beads from the Middle Stone Age layers of Sibudu Cave, South Africa. *J. Archaeol. Sci.* **35**, 2675–2685 (2008).

40. F. d'Errico, C. Henshilwood, M. Vanhaeren, K. Van Niekerk, *Nassarius kraussianus* shell beads from Blombos Cave: Evidence for symbolic behaviour in the Middle Stone Age. *J. Hum. Evol.* **48**, 3–24 (2005).

41. C. S. Henshilwood, F. D'errico, C. W. Marean, R. G. Milo, R. Yates, An early bone tool industry from the Middle Stone Age at Blombos Cave, South Africa: Implications for the origins of modern human behaviour, symbolism and language. *J. Hum. Evol.* **41**, 631–678 (2001).

42. T. Hodgskiss, An investigation into the properties of the ochre from Sibudu, KwaZulu-Natal, South Africa. *South. Afr. Humanit.* **24**, 99–120 (2012).

43. J. R. McGrath, B. L. MacDonald, D. Stalla, Middle Stone Age mineral pigment procurement at Pinnacle Point 5–6 North, Western Cape Province, South Africa. *Archaeometry* **64**, 193–217 (2022).

44. L. Dayet, F.-X. Le Bourdonnec, F. Daniel, G. Porraz, P.-J. Texier, Ochre provenance and procurement strategies during the Middle Stone Age at Diepkloof Rock Shelter, South Africa. *Archaeometry* **58**, 807–829 (2016).

45. M. Lombard, Direct evidence for the use of ochre in the hafting technology of Middle Stone Age tools from Sibudu Cave. *South. Afr. Humanit.* **18**, 57–67 (2006).

46. M. White, N. Ashton, D. Bridgland, Twisted handaxes in middle pleistocene britain and their implications for regional-scale cultural variation and the deep history of acheulean hominin groups. *Proc. Prehist. Soc.* **85**, 61–81 (2019).

47. T. A. Sumner, A refitting study of late Early to Middle Stone Age lithic assemblages from the site of Kudu Koppie, Limpopo Province, South Africa. *J. Afr. Archaeol.* **11**, 133–153 (2013).

48. D. E. Rosso, F. d'Errico, A. Queffelec, Patterns of change and continuity in ochre use during the late Middle Stone Age of the Horn of Africa: The Porc-Epic Cave record. *PLOS ONE* **12**, e0177298 (2017).

49. I. Watts, The pigments from Pinnacle Point Cave 13B, Western Cape, South Africa. *J. Hum. Evol.* **59**, 392–411 (2010).

50. F. d'Errico, M. Vanhaeren, "Microscopic and technological analysis of decorated ochre crayons from Piekary IIa, layer 6. Implications for the emergence of symbolism in Europe," in *Middle and Early Upper Palaeolithic of the Krakow Region. Piekary IIa*, vol. 6, Musées Royaux d'Art et d'Histoire, Monographie de Préhistoire Générale, Bruxelles (2008), pp. 149–160.

51. F. d'Errico, A. Nowell, A new look at the Berekhat Ram figurine: Implications for the origins of symbolism. *Camb. Archaeol. J.* **10**, 123–167 (2000).

52. D. E. Bar-Yosef Mayer, Towards a typology of stone beads in the Neolithic Levant. *J. Field Archaeol.* **38**, 129–142 (2013).

53. K. Douze, S. Wurz, C. S. Henshilwood, Techno-cultural characterization of the MIS 5 (c. 105–90 Ka) lithic industries at Blombos Cave, Southern Cape, South Africa. *PLOS ONE* **10**, e0142151 (2015).

54. C. Tribolo, N. Mercier, M. Selo, H. Valladas, J.-L. Joron, J.-L. Reyss, C. Henshilwood, J. Sealy, R. Yates, TL dating of burnt lithics from Blombos Cave (South Africa): Further evidence for the antiquity of modern human behaviour. *Archaeometry* **48**, 341–357 (2006).

55. M. M. Haaland, C. E. Miller, O. F. Unhammer, J. P. Reynard, K. L. van Niekerk, B. Ligouis, S. M. Mentzer, C. S. Henshilwood, Geoarchaeological investigation of occupation deposits in Blombos Cave in South Africa indicate changes in site use and settlement dynamics in the Southern Cape during MIS 5b-4. *Quatern. Res.* **100**, 170–223 (2021).

56. J. A. Malan, "The stratigraphy and sedimentology of the Bredasdorp Group, Southern Cape Province," thesis, University of Cape Town, Rondebosch (1990).

57. C. S. Henshilwood, J. C. Sealy, R. Yates, K. Cruz-Uribe, P. Goldberg, F. E. Grine, R. G. Klein, C. Poggenpoel, K. Van Niekerk, I. Watts, Blombos Cave, Southern Cape, South

Africa: Preliminary report on the 1992–1999 excavations of the Middle Stone Age levels. *J. Archaeol. Sci.* **28**, 421–448 (2001).

58. M. Lombard, Evidence for change in Middle Stone Age hunting behaviour at Blombos Cave: Results of a macrofracture analysis. *S. Afr. Archaeol. Bull.* **62**, 62–67 (2007).

59. S. Soriano, P. Villa, A. Delagnes, I. Degano, L. Pollarolo, J. J. Lucejko, C. Henshilwood, L. Wadley, The Still Bay and Howiesons Poort at Sibudu and Blombos: Understanding Middle Stone Age technologies. *PLOS ONE* **10**, e0131127 (2015).

60. C. Henshilwood, F. d'Errico, M. Vanhaeren, K. van Niekerk, Z. Jacobs, Middle Stone Age shell beads from South Africa. *Science* **304**, 404–404 (2004).

61. F. d'Errico, C. S. Henshilwood, Additional evidence for bone technology in the southern African Middle Stone Age. *J. Hum. Evol.* **52**, 142–163 (2007).

62. F. d'Errico, L. Doyon, S. Zhang, M. Baumann, M. Lázničková-Galetová, X. Gao, F. Chen, Y. Zhang, The origin and evolution of sewing technologies in Eurasia and North America. *J. Hum. Evol.* **125**, 71–86 (2018).

63. F. d'Errico, C. Henshilwood, P. Nilssen, An engraved bone fragment from c. 70,000-year-old Middle Stone Age levels at Blombos Cave, South Africa: Implications for the origin of symbolism and language. *Antiquity* **75**, 309–318 (2001).

64. M. M. Haaland, A. M. Strauss, E. C. Velliky, S. M. Mentzer, C. E. Miller, K. L. van Niekerk, C. S. Henshilwood, Hidden in plain sight: A microanalytical study of a Middle Stone Age ochre piece trapped inside a micromorphological block sample. *Geoarchaeology* **36**, 283–313 (2021).

65. K. Tylén, R. Fusaroli, S. Rojo, K. Heimann, N. Fay, N. N. Johannsen, F. Riede, M. Lombard, The evolution of early symbolic behavior in *Homo sapiens*. *Proc. Natl. Acad. Sci. U.S.A.* **117**, 4578–4584 (2020).

66. E. Mellet, I. Colagè, A. Bender, C. S. Henshilwood, K. Hugdahl, T. C. Lindstrøm, F. d'Errico, What processes sparked off symbolic representations? A reply to Hodgson and an alternative perspective. *J. Archaeol. Sci. Rep.* **28**, 102043 (2019).

67. J.-B. Mallye, C. Thiébaut, V. Mourre, S. Costamagno, É. Claud, P. Weisbecker, The Mousterian bone retouchers of Noisetier Cave: Experimentation and identification of marks. *J. Archaeol. Sci.* **39**, 1131–1142 (2012).

68. R. Blasco, J. Rosell, F. Cuartero, J. F. Peris, A. Gopher, R. Barkai, Using bones to shape stones: MIS 9 bone retouchers at both edges of the Mediterranean Sea. *PLOS ONE* **8**, e76780 (2013).

69. L. Doyon, Z. Li, H. Li, F. d'Errico, Discovery of circa 115,000-year-old bone retouchers at Lingjing, Henan, China. *PLOS ONE* **13**, e0194318 (2018).

70. C. Verna, F. d'Errico, The earliest evidence for the use of human bone as a tool. *J. Hum. Evol.* **60**, 145–157 (2011).

71. H. G. Nami, V. G. Scheinsohn, L. Hannus, L. Rossum, R. Winhan, in *Proceedings of the 1993 Bone Modification Conference*, Archeology Laboratory, Augustana College (1997), pp. 256–257.

72.. David, M. Sørensen, First insights into the identification of bone and antler tools used in the indirect percussion and pressure techniques during the early postglacial. *Quat. Int.* **423**, 123–142 (2016).

73. M. Baumann, S. Hinguant, The Solutrean bone industry from Rochefort Cave (Saint-Pierre-sur-Erve, Mayenne, France). *PALEO Rev. Archéol. Préhist.* , 43–63 (2016).

74. E. Kashina, A. Simonenko, M. Zhilin, Pressure flakers of late neolithic forest hunter-gatherer-fishers of Eastern Europe and their remote counterparts. *Open Archaeol.* **9**, 20220349 (2023).

75. J. M. Beretta, M. A. Zubimendi, ¿Qué sería de la industria lítica sin ellos?: Retocadores de hueso en la costa norte de Santa Cruz, Argentina. *Atek Na [En la tierra]* **5**, 85–123 (2015).

[What would the lithic industry be without them?: Bone retouchers on the north coast of Santa Cruz, Argentina].

76. T. Hodgskiss, L. Wadley, How people used ochre at Rose Cottage Cave, South Africa: Sixty thousand years of evidence from the Middle Stone Age. *PLOS ONE* **12**, e0176317 (2017).

77. T. Hodgskiss, Ochre use in the Middle Stone Age at Sibudu, South Africa: Grinding, rubbing, scoring and engraving. *J. Afr. Archaeol.* **11**, 75–95 (2013).

78. J. R. McGrath, "Human signaling ecology: A case study of late Pleistocene mineral pigment assemblages from southernmost Africa," thesis, University of Iowa, Iowa City, IA (2020).

79. U. Evans, Hollow rock shelter, a Middle Stone Age site in the Cederberg. *South. Afr. Field Archaeol.* **3**, 63–73 (1994).

80. M. Lombard, A. Högberg, L. Wadley, Temporal perspectives on Still Bay point production at Sibudu Cave, KwaZulu-Natal, in the context of southern Africa. *Azania Archaeol. Res. Africa* **54**, 141–176 (2019).

81. L. Wadley, Partners in grime: Results of multi-disciplinary archaeology at Sibudu Cave. *South. Afr. Human.* **18**, 315–341 (2006).

82. M. Lombard, A. Högberg, The Still Bay points of Apollo 11 Rock Shelter, Namibia: An inter-regional perspective. *Azania Archaeol. Res. Africa* **53**, 312–340 (2018).

83. C. S. Henshilwood, C. W. Marean, The origin of modern human behavior. *Curr. Anthropol.* **44**, 627–651 (2003).

84. C. S. Henshilwood, "Fully symbolic sapiens behaviour: Innovation in the Middle Stone Age at Blombos Cave, South Africa," in *Rethinking the Human Revolution: New Behavioural and Biological Perspectives on the Origin and Dispersal of Modern Humans*, C. Stringer, P. Mellars, Eds. (University of Cambridge Press, 2007), pp. 123–132.

85. C. S. Henshilwood, B. Dubreuil, “Reading the artifacts: Gleaning language skills from the Middle Stone Age in southern Africa,” in *The Cradle of Language*, R. Botha, C. Knight, Eds. (Oxford Univ. Press, 2009), pp. 41–61.

86. P. Wiessner, Style and social information in Kalahari San projectile points. *Am. Antiq.* **48**, 253–276 (1983).

87. P. Mellars, “Symbolism, language, and the neanderthal mind,” in *Modelling the Early Human Mind* (McDonald Institute for Archaeological Research, 1996), pp. 15–32.

88. C. W. Marean, M. Bar-Matthews, E. Fisher, P. Goldberg, A. Herries, P. Karkanas, P. J. Nilssen, E. Thompson, The stratigraphy of the Middle Stone Age sediments at Pinnacle Point Cave 13B (Mossel Bay, Western Cape Province, South Africa). *J. Hum. Evol.* **59**, 234–255 (2010).

89. T. Hodgskiss, Identifying grinding, scoring and rubbing use-wear on experimental ochre pieces. *J. Archaeol. Sci.* **37**, 3344–3358 (2010).

90. F. d’Errico, The invisible frontier. A multiple species model for the origin of behavioral modernity. *Evol. Anthropol.* **12**, 188–202 (2003).
